# Integrated analysis of insulin resistance reveals metabolic remodeling following diet switch–triggered calorie reduction

**DOI:** 10.1126/sciadv.aed0535

**Published:** 2026-05-06

**Authors:** Xiaowen Duan, Lucy M. Davis, Satish Patel, Guillaume Bidault, Lu Long, Benjamin Jenkins, Yao Yi, Pushpa Pushpa, Julia R. Wesseling, Albert Koulman, Antonio Vidal-Puig, Daniel J. Fazakerley, David B. Savage

**Affiliations:** ^1^ University of Cambridge Metabolic Research Laboratories, Wellcome Trust-MRC Institute of Metabolic Science, Cambridge CB2 0QQ, UK.; ^2^ Department of Medicine, School of Clinical Medicine, University of Cambridge, Cambridge CB2 0AW, UK.

## Abstract

Insulin resistance (IR) links obesity to metabolic disorders. While weight loss reverses IR, we show that calorie reduction (CR) can do so in obese mice within a day before significant weight loss. In contrast to whole-body IR, individual tissues do not revert to their original chow diet–fed states after CR. In the liver, improved insulin sensitivity correlates with reduced triacylglycerol and diacylglycerol and protein kinase C epsilon activity, alongside substantially decreased de novo lipogenesis and increased ketones. In muscle, insulin-sensitive glucose disposal was restored, whereas obesity-associated adipose tissue changes largely persisted following CR, specifically the reduction in fasting lipolytic activity mediated at least, in part, by lower β-adrenergic receptor 3 expression. This, combined with enhanced oxidative pathways in muscle and liver, resulted in lowered plasma free fatty acid levels and muscle and liver lipids, facilitating insulin-stimulated glucose disposal and thus restored insulin sensitivity.

## Teaser

Integrated insights into mechanisms driving acute reversal of insulin resistance enhance understanding of the condition.

## INTRODUCTION

Approximately 537 million adults currently have diabetes worldwide, with about 90% of these having type 2 diabetes (T2D) (*1*). Insulin resistance (IR) is present in >80% of patients with T2D (*2*) and underpins the association of obesity with T2D. Recent data suggest that as many as 40% of young American nondiabetic adults (aged 18 to 44 years) are insulin resistant (*3*). In addition to significantly increasing the risk of developing prediabetes and overt T2D, IR is strongly associated with metabolic dysfunction–associated steatotic liver disease and cardiovascular disease risk, even in the absence of diabetes. Therefore, understanding and reversing IR remain a global health priority.

Obesity, particularly central adiposity, is the predominant cause of IR. Weight loss very effectively alleviates IR, with insulin-resistant diabetes being treated and even reversed by bariatric surgery (*4*–*6*), low-energy diets (*7*), and weight loss inducing GLP1 agonists (*8*, *9*). Multiple mechanisms are proposed to explain why obesity is so strongly associated with IR. These include the adipose expandability hypothesis and lipotoxic effects of ectopic fat accumulation (*10*–*12*), diacylglycerol (DAG)–mediated activation of novel protein kinase Cs (PKCs) (*13*–*15*), a role for ceramides (*16*–*18*), endoplasmic reticulum (ER) stress (*19*, *20*), reactive oxygen species (*21*–*24*) and reductive stress (*25*, *26*), and inflammatory cytokines (*27*–*29*). Hyperinsulinaemia is also well known to induce IR (*30*–*32*), and interesting recent findings suggest that obesity is associated with hyperinsulinaemia even in the absence of IR (*33*). However, the potential for reversing IR independently of reducing obesity and the comprehensive physiological changes accompanying the restoration of insulin sensitivity remain largely unexplored.

To date, most studies focused on understanding IR were either cross sectional or prospective in nature. Here, we describe a mouse model in which obesity-associated IR is very rapidly (within as little as 1 to 3 days) reversed in response to reduced calorie intake, independent of obesity reversal. In addition to the basic and translationally important insight that IR can be reversed as rapidly as this, this model provides an opportunity to understand the integrated physiological changes occurring across all insulin-targeting tissues during the dynamic development and reversal of IR.

## RESULTS

### IR is acutely reversed by reduced caloric intake before the reversal of obesity

**Fig. 1.**
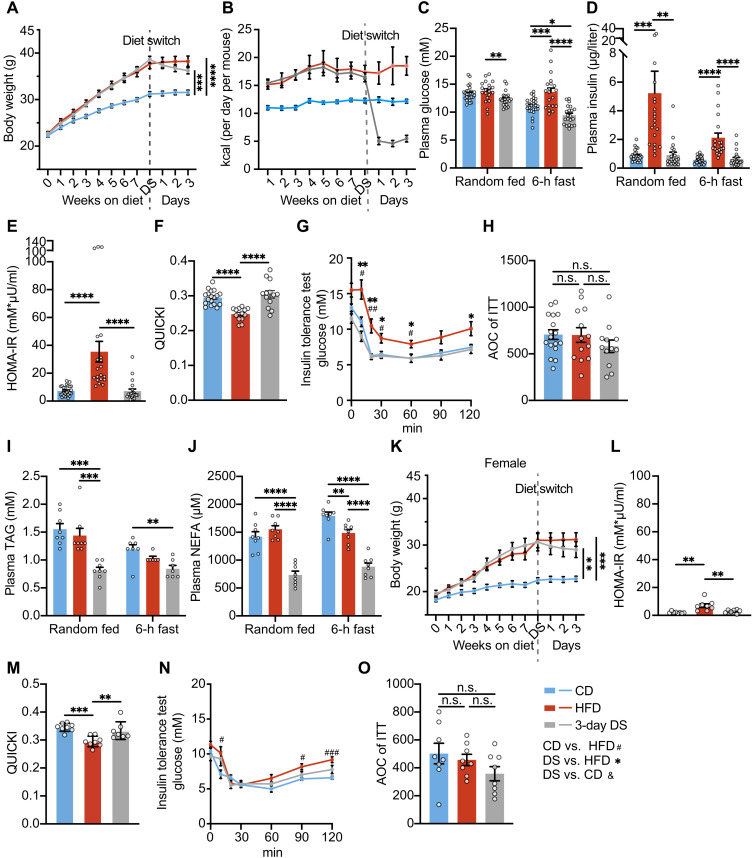
IR is acutely reversed by reduced caloric intake before body weight normalization in both male and female mice. (**A** to **J**) Body weight (A), food intake (B), plasma glucose (C), plasma insulin (D), HOMA-IR (E), QUICKI (F), insulin tolerance test (ITT) (G), area of the curve (AOC) of ITT (H), plasma TAG (I), and plasma nonesterified fatty acid (NEFA) (J) of male mice fed with CD, HFD, or HFD followed by switching back to CD for 3 days [diet switch (DS)]. Insulin was administered at a dose of 1 U/kg of body weight for the ITT. [*n* = 5 for (B), *n* = 6 to 8 for (I) and (J), and *n* = 13 to 26 for others]. h, hour. (**K** to **O**) Body weight (K), HOMA-IR (L), QUICKI (M), ITT (N), and AOC of ITT (O) of female mice fed with CD, HFD, or HFD followed by switching back to CD for 3 days. Insulin was administered at a dose of 0.5 U/kg of body weight for the ITT. (*n* = 8). Error bars represent mean ± SEM. *n* number denotes the number of cages in (B) and biological replicates for other figures. Significance for (A), (C) and (D), (E) and (F), (H) to (J), (K) to (M), and (O) was determined using one-way analysis of variance (ANOVA) with Tukey test. Significance for (A) and (K) was calculated on the basis of the body weight at the end of the study. Significance for (G) and (N) was calculated for each time point, using mixed-effects analysis with Tukey test, (#) comparison between CD and HFD groups, (*) comparison between DS and HFD groups, and (&) comparison between DS and CD groups. Not significant (n.s.), *P* > 0.05, *P < 0.05, **P < 0.01, ***P < 0.001, and ****P < 0.0001.

Male C57BL/6 mice fed a high-fat diet (HFD) for 8 weeks displayed a sustained increase in energy intake and significant weight gain (Fig. 1, A and B). This is not associated with a change in random-fed circulating glucose concentrations, but a 6-hour fasting glucose was elevated in the HFD-fed mice (Fig. 1C). HFD feeding led to a marked increase in both fed and fasting insulin concentrations (Fig. 1D), with a corresponding rise in homeostatic model assessment for insulin resistance (HOMA-IR) and a reduction in the quantitative insulin sensitivity check index (QUICKI) (Fig. 1, E and F).

Upon switching from HFD to chow diet (CD) [diet switch (DS)], mice spontaneously reduced energy intake by 70%, maintaining this reduction for at least 3 days (Fig. 1B). This led to marginal weight loss (2.37 g; 6.14%) as expected (Fig. 1A). However, IR as assessed by fasting glucose and insulin (Fig. 1, C and D), HOMA-IR (Fig. 1E), QUICKI (Fig. 1F), and an insulin tolerance test (ITT) (Fig. 1G) was fully restored to levels comparable to CD-fed controls well before weight returned to that of the CD-fed group. Area of the curve (AOC) analysis of the ITT suggests that the reduction of glucose was similar in all three groups (Fig. 1H), although this is in the context of insulin injection in mice with very different fasting or baseline insulin concentrations (Fig. 1D). These observations are similar as early as 1 day after DS (weight loss of 0.84 g; 2.2%) (fig. S1, A to G) and also if one reduces energy intake to a similar degree (70% reduction) in mice maintained on an HFD (weight loss of 1.66 g; 3.92%) (fig. S1, H to N). These data suggest that the key intervention is the reduction in energy intake rather than the change in macronutrient intake. HFD feeding for 8 weeks had a very modest impact on circulating triacylglycerol (TAG) and nonesterified fatty acid (NEFA) concentrations, but DS was associated with significant reductions in both fed and fasted TAG and NEFA levels (Fig. 1, I and J).

Female C57BL/6 mice are less prone to HFD-induced IR, as indicated by lower plasma insulin levels, HOMA-IR, and higher QUICKI compared to male mice (Fig. 1, K and N, and fig. S1, O and P). However, 3-day DS (led to weight loss of 1.59 g; 5.19%) similarly restored both fed and fasting insulin levels, as well as HOMA-IR and QUICKI, to the level of CD-fed controls (Fig. 1, L and M, and fig. S1P). AOC analysis was similar in all groups as noted in the male mice (Fig. 1O).

### Insulin-stimulated 2-deoxyglucose clearance and glucose uptake index

**Fig. 2.**
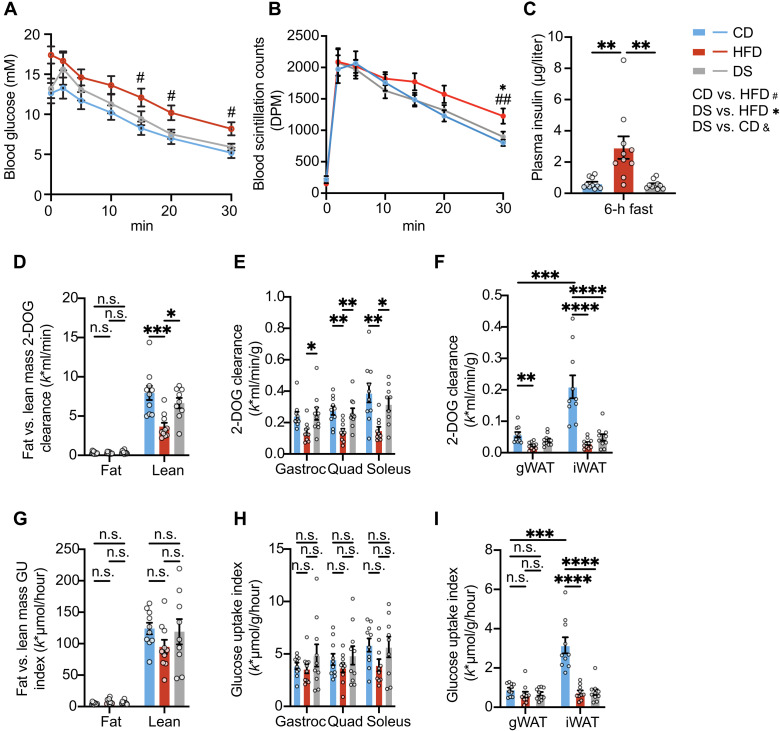
Tissue-specific insulin-stimulated glucose disposal in mice under anesthesia. (**A** and **B**) Blood glucose levels (A) and scintillation counts (B) across the course of a radioisotope-labeled ITT in male mice fed with CD, HFD, or HFD followed by switching back to CD for 3 days (DS). Insulin was administered at a dose of 0.75 U/kg of the average body weight of CD group mice for the ITT (*n* = 8). (**C**) Plasma insulin levels before insulin injection in this experiment (*n* = 10). (**D**) 2-DOG clearance into fat versus lean mass across the whole body, estimated on the basis of fat mass, lean mass data from EchoMRI, and 2-DOG clearance per unit of tissue from the current experiment (*n* = 10). (**E** and **F**) 2-DOG clearance in skeletal muscle (E) or adipose tissue (F) (*n* = 10). Gastroc, gastrocnemius; Quad, quadriceps. (**G**) Glucose uptake (GU) index in fat versus lean mass across the whole body, estimated on the basis of fat mass, lean mass data from EchoMRI, and GU index per unit of tissue from the current experiment (*n* = 10). (**H** and **I**) GU index in skeletal muscle (H) or adipose tissue (I) (*n* = 10). Error bars represent mean ± SEM. *n* number denotes biological replicates. Significance for (A) and (B) was calculated for each time point, using one-way ANOVA with Tukey test, (#) comparison between CD and HFD groups, (*) comparison between DS and HFD groups, and (&) comparison between DS and CD groups. Significance for (C) to (I) was determined by one-way ANOVA with Tukey test. Significance of the comparison between gonadal white adipose tissue (gWAT) and inguinal white adipose tissue (iWAT) in CD group in (F) and (I) was determined by paired *t* test. Not significant (n.s.), *P* > 0.05, *P < 0.05, **P < 0.01, ***P < 0.001, and ****P < 0.0001.

To understand where glucose was being disposed, we performed a radioisotope-labeled ITT in mice under anesthesia. In response to an intravenous insulin dose based on the average body weight of CD-fed mice, reductions in blood glucose were observed in this study (Fig. 2A), which was limited to the first 30 min after insulin injection, similar to those observed in the conventional ITT (Fig. 1G). Blood 2-deoxyglucose (2-DOG) scintillation counts were consistent with similar rates of 2-DOG clearance in the CD-fed and 3-day DS groups (Fig. 2B). Fasting insulin, measured before the ITT, was significantly higher in HFD-fed compared to CD-fed mice but was similar between CD group and DS mice (Fig. 2C). Both 2-DOG clearance and glucose uptake index were calculated using the methods previously reported (*34*). Total glucose disposal in fat mass (average fat mass of 7.3 g) was ~4.9% of that disposed in lean mass (average lean mass of 25.7 g) (Fig. 2, D and G), in keeping with published literature suggesting that skeletal muscle accounts for most of insulin-stimulated glucose disposal (*35*–*38*).

Radiolabeled 2-DOG uptake in the soleus, gastrocnemius, and quadriceps muscles was lower in the HFD-fed mice and restored to levels seen in the CD control group in the 3-day DS mice (Fig. 2E), but if one adjusts for ambient blood glucose levels that were higher in the HFD group, then “adjusted glucose disposal” is similar in all three groups in skeletal muscle (Fig. 2H). This is in the context of significantly higher fasting or baseline insulin concentrations in the HFD group (Fig. 2C). In contrast, both 2-DOG clearance and glucose uptake index in subcutaneous inguinal white adipose tissue (iWAT) were reduced in HFD-fed mice and remained similarly reduced in the 3-day DS group. However, in gonadal white adipose tissue (gWAT), both 2-DOG clearance and glucose uptake index were lower compared to iWAT from CD mice, with 2-DOG clearance reduced and glucose uptake index unchanged by HFD feeding. Neither parameter was altered by DS in gWAT (Fig. 2, F and I).

### A DS-induced elevation in FA oxidation contributes to reduced intramuscular lipids

**Fig. 3.**
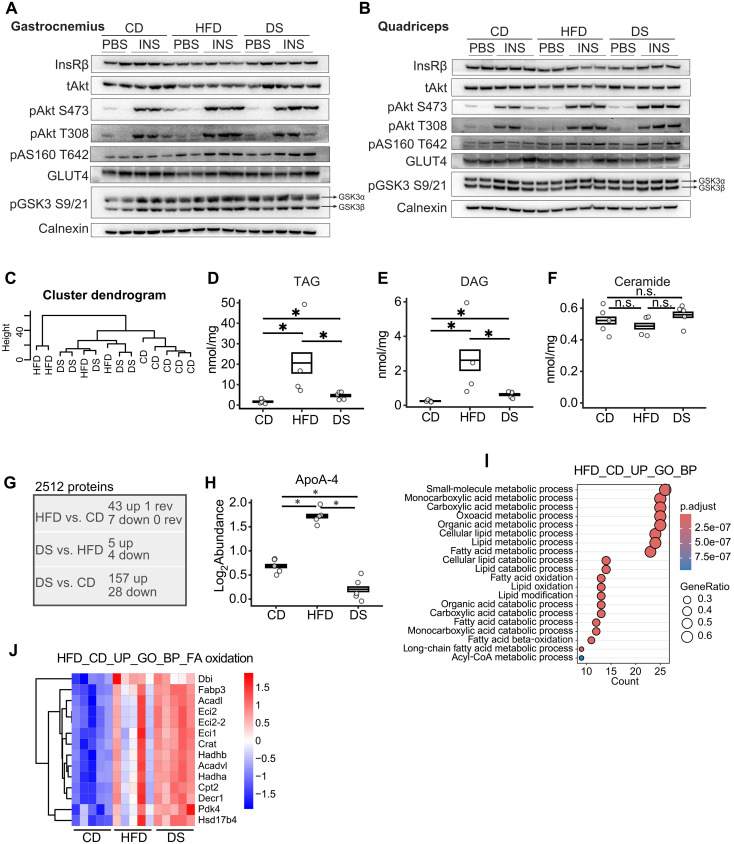
Three-day DS elevates FA oxidation and reduces intramuscular lipids. (**A** and **B**) Western blots for selected proximal components of the insulin signaling pathway in the gastrocnemius (A) and quadriceps muscle (B) from male mice fed with CD, HFD, or HFD followed by switching back to CD for 3 days (DS). Insulin was administered at a dose of 1 U/kg of the average body weight of CD group mice. INS, insulin. (**C**) Hierarchical cluster analysis using lipidomics data from quadriceps muscle. (**D** to **F**) Concentrations of TAG (D), DAG (E), and ceramide (F) in quadriceps muscle (*n* = 4 to 5). (**G**) Numbers of significantly regulated proteins (defined as adjusted *P* ≤ 0.05) in indicated comparisons based on proteomics analyses in gastrocnemius muscle. “rev” implies reversal to CD state. (**H**) Log_2_Abundance of apolipoprotein A-4 (apoA-4) across diet groups based on proteomics data in gastrocnemius muscle. (**I**) Pathway analysis of proteins significantly up-regulated by HFD feeding compared to CD control in the proteomics data using gene ontology_biological process (GO_BP) database. (**J**) Heatmap displaying the expression of proteins included in the “fatty acid oxidation” pathway in (I). Box plots represent mean ± SEM. *n* number for (C) to (H) denotes biological replicates. Significance for (D) to (H) was determined using limma package in R. Not significant (n.s.), *P* > 0.05, and *P < 0.05.

In skeletal muscle, minimal differences were detected in proximal insulin signaling components, including insulin receptor (InsR) expression and Akt phosphorylation at both the Ser473 and Thr308 sites, as well as in the downstream targets of Akt, including AS160 and glycogen synthase kinase 3β (GSK3β; Fig. 3, A and B, and fig. S2, A and B). This observation is consistent with previous studies showing minimal change in proximal insulin signaling in muscle (*39*, *40*). Glucose transporter type 4 (GLUT4) expression appeared to be reduced in the HFD group in the gastrocnemius, but this was not seen in quadriceps muscle and was not reversed in the 3-day DS mice (Fig. 3, A and B, and fig. S2, A and B). Together with the 2-DOG clearance and glucose uptake results in Fig. 2, these data suggest that insulin action in skeletal muscle is largely retained in the HFD-fed mice, but this requires higher concentrations of insulin in the blood. Following DS, the level of insulin required for similar signaling and glucose disposal is restored to that seen in CD controls.

Ectopic lipid accumulation has been widely implicated in causing muscle IR (*41*), therefore lipidomic analyses in muscle were performed. Twenty-two lipid families (fig. S3A) consisting of 400 individual lipid species were identified. Unsupervised clustering separated out CD-fed mice but did not separate the HFD and the 3-day DS groups (Fig. 3C). Both TAG and DAG were higher in HFD-fed mice and then significantly lower after 3-day DS although still elevated compared to the CD group (Fig. 3, D and E, and fig. S3, B to D). The other lipid family with similar profiles was MGDG (monogalacto-DAG) (fig. S3A). Some lipid families were higher in HFD-fed mice but then remained elevated in the 3-day DS group, including phosphatidyl-glycerol (fig. S3A). There were no changes in total ceramide content (Fig. 3F and fig. S3E).

Proteomic analysis of muscle samples identified 2512 proteins, but expression of very few proteins was altered (*n* = 50) in the HFD-fed mice. Abundance of only one of these 50 proteins was reversed by 3-day DS, ApoA-4 (Fig. 3, G and H), an apolipoprotein escorting chylomicrons and high-density lipoprotein and regulating cholesterol efflux. A higher number of proteins were differentially expressed between the 3-day DS and CD groups (*n* = 185) (Fig. 3G), but unsupervised clustering did not separate out the three groups (fig. S4A). Pathway analyses using significantly altered proteins suggested that FA oxidation was elevated in HFD-fed mice and was further increased by 3-day DS (Fig. 3, I and J, and fig. S4, B and C), suggesting a metabolic shift toward the utilization of FA as the primary energy source in the muscle.

### DAG, PKCε, and proximal insulin signaling changes were reversed after 3-day DS in the liver

**Fig. 4.**
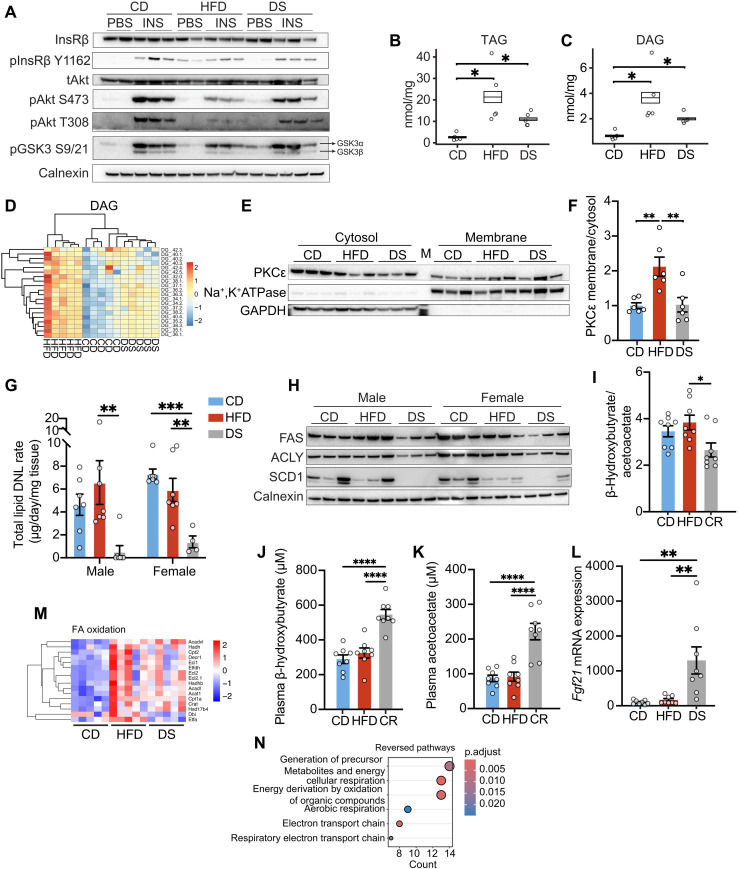
DAG, PKCε, and proximal insulin signaling changes were reversed after DS in the liver. (**A**) Expression of selected insulin signaling components in the liver from male mice fed with CD, HFD, or HFD followed by DS for 3 days. Insulin was administered at a dose of 1 U/kg of the average body weight of CD mice. (**B** and **C**) Concentrations of TAG (B) and DAG (C) (*n* = 5). (**D**) Levels of all DAG species detected in lipidomics analyses, and the hierarchical clustering across groups. (**E**) PKCε expression in cytosolic and membranous fractions of the liver. M, molecular weight marker lane. (**F**) Quantification of (E); subcellular PKCε expression was normalized to Na^+^- and K^+^-dependent adenosine triphosphatase (ATPase) (Na^+^,K^+^-ATPase) or glyceraldehyde-3-phosphate dehydrogenase (GAPDH) before the calculation of the ratio (*n* = 6). (**G**) DNL rate (*n* = 4 to 7). (**H**) Expression of DNL proteins. (**I** to **K**) Plasma concentrations of β-hydroxybutyrate (BHB) (J), acetoacetate (AcAc) (K), and their ratio (I) in male mice fed with CD, HFD, or those after CR by 70% on HFD for 18 hours (*n* = 8). (**L**) Relative mRNA expression of *Fgf21* (*n* = 8). (**M**) Heatmap displaying the expression of proteins included in the “fatty acid β-oxidation using acyl-CoA dehydrogenase” pathway in (fig. S7C). (**N**) Pathway analysis of proteins significantly regulated by HFD feeding versus CD controls and reversed by 3-day DS in the liver proteomics data using GO_BP database. The color gradient for heatmaps represents the *z*-score values, with red color indicating higher and blue indicating lower values. Box plots represent mean ± SEM. *n* number denotes biological replicates. Significance for (B) and (C) was determined using limma package in R. Significance for (F) and (G) and (I) to (L) was determined using one-way ANOVA with Tukey test. Not significant (n.s.), *P* > 0.05, *P < 0.05, **P < 0.01, ***P < 0.001, and ****P < 0.0001.

The liver plays a particularly important role in regulating the fasting metabolic state. The significant elevation of HOMA-IR, derived from fasting glucose and insulin, in HFD-fed mice (Fig. 1E) suggests pronounced hepatic IR. In keeping with these data, insulin-stimulated Akt phosphorylation at both the Ser473 and Thr308 sites was significantly reduced in livers of the obese mice (Fig. 4A and fig. S5A). Corresponding changes were also apparent for GSK3 phosphorylation, although these are more subtle (Fig. 4A and fig. S5A). The changes in Akt and GSK3β phosphorylation were reversed in the 3-day DS group (Fig. 4A and fig. S5A). InsR expression was modestly reduced (Fig. 4A and fig. S5A), but basal-state InsR Tyr1162 phosphorylation was elevated in HFD-fed mice, with a nonsignificant response to insulin stimulation, whereas the increment was significant in CD-fed control mice (Fig. 4A and fig. S5A). In the DS group, basal-state InsR Tyr1162 phosphorylation was similar to the CD group, and the response to insulin was partially restored (Fig. 4A and fig. S5A).

Lipidomic analyses identified 20 lipid families consisting of 366 individual lipid species. Unsupervised clustering distinguished the three groups (fig. S6A); both total hepatic TAG and DAG were elevated in the HFD group and tended to fall in the 3-day DS group (Fig. 4, B and C, and fig. S6B). Some lipid families were higher in HFD-fed mice and then significantly reduced in the 3-day DS group, including Monolysocardiolipin, lysophosphatidylcholine, MGDG, and phosphatidyl-serine (fig. S6B).

Although total DAG levels were still significantly higher in the 3-day DS than in the CD group, cluster analysis based on individual DAG species revealed greater similarity between the 3-day DS and CD groups rather than the HFD group. This contrasts with the patterns observed for TAG and ceramide (Fig. 4D and fig. S6, D and E). Total ceramides were again largely unchanged (fig. S6, B and C), but ceramides 36:0, 38:1, and 39:1 were elevated in the HFD group and reversed by 3-day DS (fig. S6F). One ceramide species 44:2 was reduced by HFD feeding and increased after the 3-day DS (fig. S6F).

PKCε activation has been strongly associated with DAG accumulation in this context (*13*). The membrane-to-cytosol ratio of PKCε was ~2-fold higher in HFD mice (Fig. 4, E and F), and this change was reversed following the 3-day DS (Fig. 4, E and F). Enhanced ER stress has been reported in the liver in obese mice and implicated in causing hepatic IR (*19*). However, Eif2α phosphorylation was substantially increased following the 3-day DS in our study, suggesting that activation of the integrated stress response in this context at least is not sufficient to induce IR (fig. S6G).

De novo lipogenesis (DNL) is a key insulin-responsive metabolic pathway in the liver so we proceeded to evaluate it in our model. Although the HFD-fed mice were insulin resistant, the rate of hepatic DNL was similar to that of CD-fed controls (Fig. 4G). Expression of lipogenic enzymes, including FA synthase (FAS) and adenosine 5′-triphosphate (ATP) citrate lyase (ACLY), was sustained on an HFD, while expression of a key desaturase, stearoyl–coenzyme A (CoA) desaturase 1 (SCD1), was reduced (Fig. 4H). Reducing calorie intake via 3-day DS resulted in a substantial reduction in DNL in both male and female mice (Fig. 4G), and we noted corresponding changes in the expression of FAS, ACLY and SCD1 (Fig. 4H), supported by proteomics data (fig. S7I). The changes in DNL are also likely to be related to changes in DNL substrate availability (*42*), as DNL is very low in DS animals despite restoration of insulin signaling.

Next, the β-hydroxybutyrate (BHB)/acetoacetate (AcAc) ratio, which has been used as an index of reductive stress, was evaluated (*43*). While it was not increased in the HFD-fed mice, it was modestly reduced in the calorie-restricted mice (Fig. 4I). More notable was the increase in absolute concentrations of both BHB and AcAc (Fig. 4, J and K), suggesting enhanced ketogenesis in the mice following reduced calorie intake. In keeping with this observation, *Fgf21* mRNA expression was very significantly elevated in the 3-day DS group (Fig. 4L). In addition, expression of ketogenic enzymes was elevated in both HFD and DS groups based on proteomics data (fig. S7K), although this does not necessarily indicate their activity.

Proteomic analysis identified 5003 proteins in liver samples (fig. S7A). Two hundred ninety-three of them were differentially expressed between the HFD and CD groups, 524 between the 3-day DS and HFD groups, and 478 between the 3-day DS and CD groups (fig. S7A), but unsupervised clustering was unable to distinguish the three groups (fig. S7B). Pathway analyses suggested increased nutrient catabolism (fig. S7, C and D) and decreased lipid or steroid anabolism (fig. S7, E and F) in HFD-fed mice. Although FA oxidation protein expression tended (although this was not statistically significant) to be increased in the HFD and 3-day DS groups (Fig. 4M), mitochondrial oxidative phosphorylation was down-regulated in the 3-day DS group (Fig. 4N and fig. S7, G, H, and J). These data suggest that there may be a reduced flux through the tricarboxylic acid cycle and electron transport chain despite the catabolic state in DS mice, potentially in keeping with enhanced ketogenesis.

### Baseline lipolytic activity remained suppressed after DS, likely due to sustained reduction of β-adrenergic receptor 3 expression

**Fig. 5.**
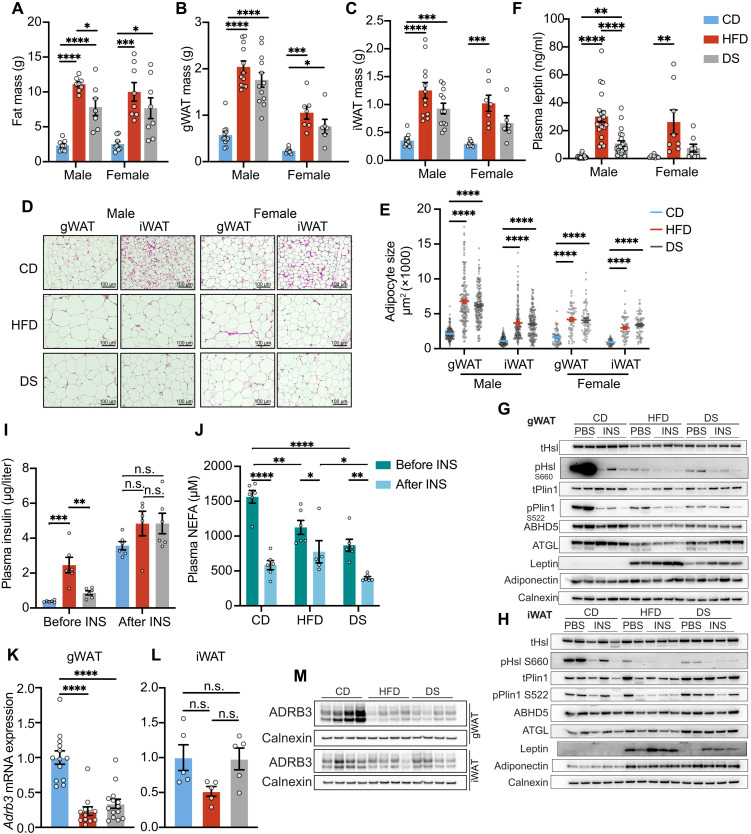
Baseline lipolytic activity remained suppressed after 3-day DS due to sustained reduction of ADRB3 expression. (**A** to **C**) Whole-body fat mass (A), gWAT mass (B), and iWAT mass (C) of male and female mice fed with CD, HFD, or HFD followed by DS for 3 days (*n* = 7 to 20). (**D**) Representative images of hematoxylin and eosin staining of gWAT and iWAT tissues. (**E**) Adipocyte size of gWAT and iWAT (*n* = 10 to 11 for males and *n* = 3 for females). (**F**) Plasma leptin concentration of male and female mice fed with CD, HFD, or HFD followed by DS for 3 days (*n* = 7 to 20). (**G** and **H**) Western blots for selected lipolytic pathway components in the gWAT (G) or iWAT (H) from mice fasted for 6 hours with or without subsequent insulin treatment for 20 min. Insulin was administered at a dose of 1 U/kg of the average body weight of CD group mice. (**I** and **J**) Plasma insulin (I) and NEFA concentrations (J) of mice fasted for 6 hours with or without subsequent insulin treatment for 20 min (*n* = 6). Insulin was administered at a dose of 1 U/kg of the average body weight of CD group mice. (**K** and **L**) Relative mRNA expression of *Adrb3* in gWAT (K) or iWAT (L) across diet groups. (**M**) Western blots displaying the expression of ADRB3 in gWAT or iWAT across diet groups. Error bars represent mean ± SEM. *n* number denotes biological replicates. Significance for (J) was determined using two-way ANOVA with Tukey test. Significance for (A) to (C), (E) and (F), (I), and (K) and (L) was determined by one-way ANOVA with Tukey test. Not significant (n.s.), *P* > 0.05, *P < 0.05, **P < 0.01, ***P < 0.001, and ****P < 0.0001.

Although relatively little glucose is disposed of directly into WAT, it does constitute the major site for surplus energy storage. Human lipodystrophy models show that disruption of this storage is associated with IR, highlighting the importance of WAT in modulating insulin sensitivity. Whole-body fat mass increased ~4-fold in the HFD-fed male and female mice compared to CD-fed mice (Fig. 5A), whereas lean mass was similar (fig. 9J). This was associated with a significant increase in adipocyte size and fat pad weight in both the gonadal and inguinal fat pads (Fig. 5, B to E). In 3-day DS mice, fat mass tended to fall but remained significantly higher than that of the CD group (Fig. 5, A to C). Changes in plasma leptin concentrations correlated with the changes in fat mass, but the increase in response to HFD feeding and the subsequent fall in the 3-day DS group were substantially greater in magnitude (Fig. 5F). We hypothesize that the marked fall in plasma insulin in the DS group is a significant determinant of this change in leptin concentrations, as insulin is a known regulator of plasma leptin (*44*).

In relation to the changes in glucose disposal reported in Fig. 2, reductions in InsR and GLUT4 protein expression were observed in both gonadal and inguinal fat (fig. S8, A to D) in HFD-fed mice, in keeping with previous reports (*45*–*47*). However, insulin-stimulated Akt, GSK3, and AS160 phosphorylation were mostly unchanged (fig. S8, A to D). Three-day DS rescued GLUT4 expression in gWAT but not in iWAT (fig. S8, A to D), in keeping with changes in glucose disposal in Fig. 2. InsR expression was not restored in either depot (fig. S8, A to D).

DNL also occurs in WAT, but we were unable to measure the flux in male mice due to very modest changes in labeled palmitate relative to the large amount of neutral lipid in this tissue. In female mice, this pathway appears to be more active, as reflected in higher expression of key DNL enzymes (fig. S9A). In both males and females, HFD feeding was associated with reduced expression of some of these enzymes, and their levels fell further in the 3-day DS group (fig. S9A). DNL flux was reduced in the HFD-fed female mice and remained suppressed in iWAT (fig. S9B) and was suppressed even further in gWAT (fig. S9C) in the 3-day DS group.

Another key insulin-responsive pathway in WAT is the lipolytic pathway. Here, substantial changes in the regulation of hormone sensitive lipase (HSL) phosphorylation were observed (Fig. 5, G and H, and fig. S9, D and E). HSL is phosphorylated by protein kinase A (PKA) in response to adrenergic signaling and is then dephosphorylated when adenosine 3′,5′-cyclic monophosphate levels fall in response to insulin signaling. The marked reduction in HSL phosphorylation is most evident in gWAT in CD-fed mice following insulin injection after a 6-hour fast (Fig. 5G and fig. S9D). In HFD-fed mice, “fasting HSL phosphorylation” was substantially lower than in the CD group (Fig. 5G and fig. S9D). It does then fall further after insulin stimulation, but the magnitude of this change is much smaller (Fig. 5G and fig. S9D). Similar changes were seen in the inguinal fat pad (Fig. 5H and fig. S9E). The substantial reduction in “basal” (i.e., in 6-hour–fasted mice) HSL phosphorylation in the HFD-fed mice compared to the CD group was not reversed in the 3-day DS group (Fig. 5, G and H, and fig. S9, D and E) despite their substantially lower fasting insulin concentrations (Fig. 5I).

PKA also phosphorylates Perilipin 1 (Plin1), constituting another key step in stimulating lipolysis. The Plin1 phosphorylation changes observed in response to insulin corresponded to those seen in HSL phosphorylation (Fig. 5G and fig. S9D). Adipose triglyceride lipase (ATGL) and ab-hydrolase domain containing 5 (ABHD5) expressions were modestly reduced in gWAT and, to a lesser degree, in iWAT from HFD-fed mice, and this change was only partially reversed in the 3-day DS group (Fig. 5, G and H, and fig. S9, D and E).

Higher fasting insulin concentrations in the HFD group (Fig. 5I) might account for the changes in basal HSL and Plin1 phosphorylation. However, if this were the case, then one would expect HSL and Plin1 phosphorylation to be far higher in the DS group, where insulin levels are similar to the CD group. Instead, they remain relatively low and are fully suppressed after insulin treatment (Fig. 5, G and H). In vivo, the net impact of these marked changes in HSL phosphorylation is likely modulated by differences in total fat mass and insulin concentrations. Ultimately, fasting NEFA levels are only slightly higher in CD than in HFD-fed mice, and in both instances, they are suppressed following insulin injection (Fig. 5J). In the HFD group, the net impact of insulin on blood NEFA concentrations is still present but rather muted (Fig. 5J).

To further evaluate lipolysis in a more physiological setting, we fasted mice overnight (until 8:00 a.m.) before refeeding them with CD (in all groups) for 2 hours (until 10:00 a.m.). In this case, fasting glucose and insulin concentrations were higher in the HFD group but were similar following refeeding (fig. S9, F and G), as the HFD group consumed less of the CD than the other groups (fig. S9I). Fasting NEFA concentrations were highest in the CD-fed group and fell sharply in both the CD and 3-day DS groups, whereas they remained unchanged in the HFD group after refeeding (fig. S9H).

β-Adrenergic receptor 3 (ADRB3) is the most highly expressed adrenergic receptor in rodent WAT, and as has been previously reported (*48*), its mRNA and protein expression were substantially reduced in gWAT in HFD-fed mice (Fig. 5, K to M). This change was not reversed in the 3-day DS group (Fig. 5, K to M), in keeping with the changes observed in HSL regulation. The changes in iWAT were more modest, which may relate to the fact that inflammation in iWAT lags behind that in gWAT in HFD-fed mice (*49*).

Proteomic analysis of both gWAT and iWAT samples identified ~5300 proteins in each of the tissue types (figs. S10A and S11A). Compared to the CD group, 1475 proteins were changed in gWAT and 369 in iWAT in the HFD group (figs. S10A and S11A). The expression of a small portion of these altered proteins was reversed after 3-day DS. Unsupervised clustering separated CD from the HFD and DS groups but did not distinguish the latter two in gWAT (fig. S10B). In iWAT, this analysis was unable to separate the groups (fig. S11B). Pathway analyses did reveal some significant changes in gWAT and iWAT (figs. S10, C to H, and S11, C to K), but most of these were not concordant in the two depots. A mitochondrial gene expression pathway, a mitochondrial translation pathway, and an oxidative phosphorylation pathway were down-regulated in the 3-day DS group in both WAT depots (figs. S10, C to G, and S11, E and G to K). An FA biosynthesis pathway was also down in both depots in the 3-day DS groups (figs. S10H and S11, C to F), in keeping with the DNL data.

### Longer-term HFD–induced IR is incompletely reversed by short-term calorie restriction

It has previously been suggested that longer-term HFD feeding–associated IR differs from that associated with shorter-term exposure to HFD (*50*). Cell death increases in the gonadal fat pad over time on HFD, peaking at 16 weeks (*49*). Hence, we evaluated IR changes following calorie reduction (CR) in mice fed an HFD for 18 weeks.

After 8-week HFD feeding, DS consistently resulted in a spontaneous CR by ~70%. However, after 18 weeks on an HFD, the spontaneous reduction in calorie intake in response to feeding with a CD was more variable in male mice. Therefore, to ensure that we could study the effect of reduced calorie intake on 18-week HFD-fed mice, we opted to instead keep mice on the HFD but subject them to an 18-hour calorie restriction (70% reduction of their usual energy intake), similar to the data in fig. S1 (H to K).

**Fig. 6.**
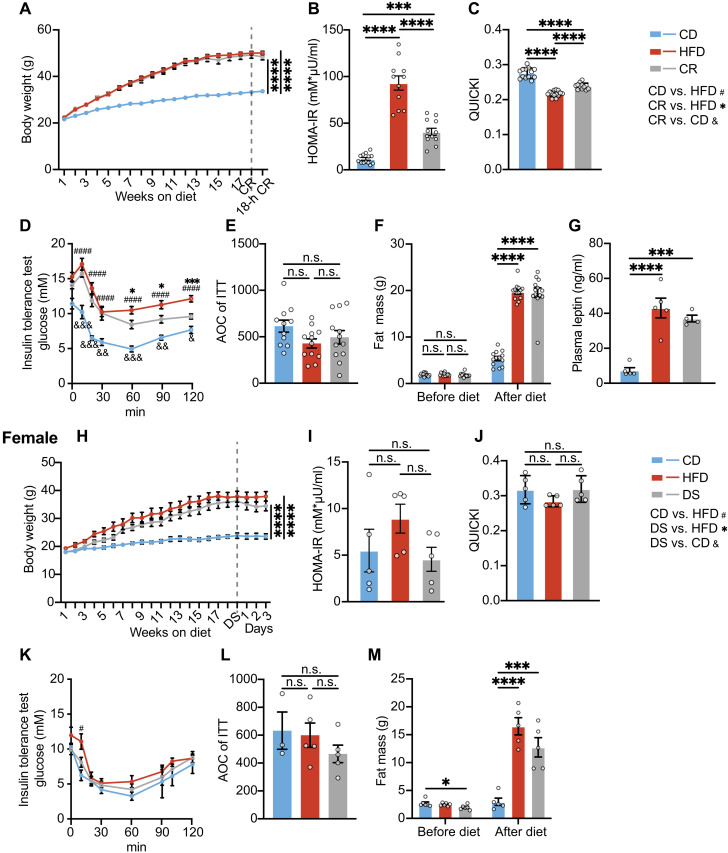
Longer-term HFD–induced IR is incompletely reversed by short-term calorie restriction. (**A** to **G**) Body weight (A), HOMA-IR (B), QUICKI (C), ITT (D), AOC of ITT (E), pre- and poststudy fat mass (F), and fasting plasma leptin (G) of male mice fed with a CD, an HFD for 18 weeks, or an HFD for 18 weeks followed by 18 hours of 70% CR while remaining on an HFD. Insulin was administered at a dose of 1 U/kg of body weight for the ITT [*n* = 11 to 13 for (A) to (F) and *n* = 4 to 5 for (G)]. (**H** to **M**) Body weight (H), HOMA-IR (I), QUICKI (J), ITT (K), AOC of ITT (L), and pre- and poststudy fat mass (M) of female mice fed with a CD for 18 weeks, an HFD for 18 weeks, or an HFD for 18 weeks followed by switching back to CD for 3 days. Insulin was administered at a dose of 0.5 U/kg of body weight for the ITT (*n* = 5). Error bars represent mean ± SEM. *n* number denotes biological replicates. Significance for (A) to (C), (E) and (F), (G) to (J), and (L) and (M) was determined using one-way ANOVA with Tukey test. Significance for (A) and (H) was calculated on the basis of the body weight at the end of the study. Significance for (D) and (K) was calculated for each time point, using mixed-effects analysis with Tukey test, (#) comparison between CD and HFD groups, (*) comparison between DS and HFD groups, and (&) comparison between DS and CD groups. Not significant (n.s.), *P* > 0.05, *P < 0.05, ***P < 0.001, and ****P < 0.0001.

When calorie restricted, body weight fell by 1.67% (0.82 g) but was not statistically significantly different from the HFD-maintained group (Fig. 6A). In this setting, fat mass and leptin did not fall significantly in the calorie-restricted group, and lean mass was also similar (Fig. 6, F and G, and fig. S12, A and B). HOMA-IR was reduced by ~50% (Fig. 6B) but remained significantly higher than that of the CD group (Fig. 6B). QUICKI decreased on HFD and was partially restored with calorie restriction (Fig. 6C). Similarly, ITT data suggested that IR was improved but not as effectively restored to that of CD-fed mice as we had observed at 8 weeks (Fig. 6D). AOC analysis was similar in all groups as noted in the mice from 8-week cohorts (Fig. 6E). In female mice fed an HFD for 18 weeks (Fig. 6, H to M), IR appeared to be alleviated after DS according to HOMA-IR and QUICKI (Fig. 6, I and J), with insignificant changes in body weight (1.38 g; 3.85%) (Fig. 6H) and a modest reduction in fat mass (Fig. 6M). However, as at 8 weeks, the females were far less insulin resistant than the equivalent male group.

**Fig. 7.**
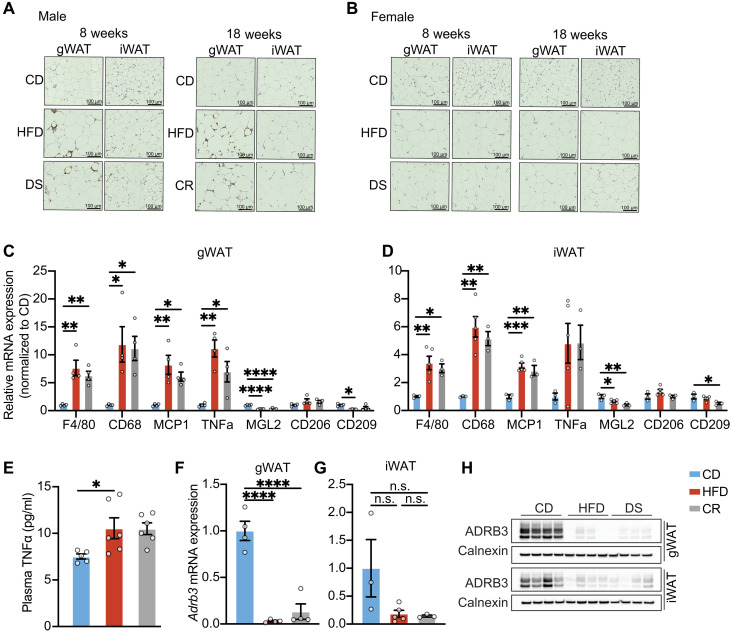
HFD-associated adipose inflammation is generally not reversed following acutely reduced calorie intake. (**A** and **B**) Representative images of histological F4/80 staining of gWAT and iWAT tissues from male (A) and female (B) mice at 8 and 18 weeks from CD, HFD, and 3-day DS/ 18-hour CR groups. (**C** and **D**) quantitative polymerase chain reaction (qPCR) data showing relative gene expression of macrophage markers (F4/80 and CD68), M1 markers [monocyte chemoattractant protein-1 (MCP1) and TNFα], and M2 markers [macrophage galactose N-acetyl-galactosamine specific lectin 2 (MGL2), CD206, and CD209] in gWAT (C) and iWAT (D) of male mice fed with a CD for 18 weeks, an HFD for 18 weeks, or an HFD for 18 weeks followed by 18 hours of 70% CR while remaining on an HFD formulation (*n* = 4). (**E**) Plasma TNFα of male mice fed with a CD for 18 weeks, an HFD for 18 weeks, or an HFD for 18 weeks followed by 18 hours of 70% CR while remaining on an HFD formulation. (**F** and **G**) Relative mRNA expression of *Adrb3* in gWAT (F) or iWAT (G) across diet groups. (**H**) Western blots displaying the expression of ADRB3 in gWAT or iWAT across diet groups. Error bars represent mean ± SEM. *n* number denotes biological replicates. Significance for (C) and (G) was determined using ordinary one-way ANOVA with Tukey test. *P < 0.05, **P < 0.01, ***P < 0.001, and ****P < 0.0001.

Existing data suggest that adipocyte death is significantly more pronounced in gWAT than in iWAT and peaks in gWAT at around 16 weeks after starting an HFD (*49*). After 18 weeks of HFD feeding, we observed a substantial increase in F4/80 immunostaining in HFD-fed mice, and this aggregated in what is now widely referred to as crown-like structures (CLSs) (Fig. 7, A and B). This did not reverse with calorie restriction in male or female mice at 8 or 18 weeks (Fig. 7, A and B). Furthermore, F4/80 staining and CLSs were more prominent after 18 weeks of HFD than after 8 weeks (Fig. 7, A and B). mRNA expression of macrophage markers (Fig. 7, C and D) and plasma tumor necrosis factor–α (TNFα) (Fig. 7E) was similarly increased in the 18-week HFD group and did not reverse with calorie restriction. Furthermore, mRNA expression of M1 and M2 macrophage markers in gWAT and iWAT does not indicate a change in macrophage polarization state with calorie restriction (Fig. 7, C and D). These data are consistent with increased adipocyte death and inflammation in HFD-fed mice as has been widely reported previously (*49*) and indicate that these changes are not reversed acutely following reduced calorie intake, suggesting that any IR reversal observed after 18 weeks with 18-hour calorie restriction is not due to changes in adipose tissue inflammation. In both gWAT and iWAT, ADRB3 mRNA and protein expression were again significantly reduced in the HFD group after 18 weeks and remained very low in the DS group (Fig. 7, F to H).

## DISCUSSION

High-fat feeding in C57BL/6 mice immediately results in increased energy intake and weight gain, and IR is apparent within a few days (*51*–*53*). Studies focused on the earliest events in this process have suggested that liver and adipose IR manifest within a week of HFD feeding, whereas muscle IR develops after ~3 weeks (*53*). In our study, 8 weeks of HFD feeding in male C57BL/6 mice significantly increased body weight, and fat mass was ~4-fold higher than CD-fed controls. This was associated with hepatic (as reflected by a marked increase in fasting glucose, insulin, and HOMA-IR) and adipose IR (as reflected by reduced glucose disposal despite higher insulin levels and changes in the regulation of lipolysis). In the case of muscle, glucose disposal and proximal insulin signaling appeared to be similar in all three groups, but this was in the presence of significantly higher insulin concentrations in the HFD group. HFD-induced changes in HOMA-IR or QUICKI and ITT were fully reversed within 3 days of the DS. This reversal was a consequence of calorie restriction and was apparent after as little as 1 day. Female mice are considerably smaller and more insulin sensitive than males, but HFD feeding is still associated with a rise in HOMA-IR and a reduction in QUICKI after 8 weeks, and this is similarly promptly reversed after the DS and reduced calorie intake.

The fact that obesity-associated IR can be so rapidly reversed despite very modest changes in body weight is unexpected but consistent with other observations in mice and humans. For example, diet switching obese HFD-fed mice to CD for 7 to 9 days has been shown to normalize glucose tolerance and hyperinsulinemia before substantial weight loss (*54*), and in diabetic rats, 3 days of a very-low–calorie diet was sufficient to reverse hyperglycaemia again before weight loss (*55*). In people with T2D, fasting glucose, insulin, and C-peptide levels decreased significantly within 1 week of bariatric surgery (*56*).

In seeking to understand how IR is so rapidly reversed, we considered skeletal muscle, as it accounts for most of insulin-stimulated glucose disposal. However, no changes in proximal insulin signaling were detected in skeletal muscle, supported by prior reports (*39*, *40*, *51*). Muscle TAG and DAG concentrations were nevertheless significantly higher in the HFD-fed mice, and these were partially reversed within 3 days of DS. It has been suggested that PKCθ activity might account for DAG-mediated muscle IR (*57*), and this could account for our observations, but several other hypotheses such as the role of reactive oxygen species also warrant further consideration (*30*, *58*).

In contrast to muscle, in the liver, proximal insulin signaling was altered, most detectable in insulin-stimulated Akt phosphorylation at both the S473 and T308 sites, and these changes were reversed following reduced calorie intake. TAG and DAG concentrations also changed in accordance with these changes as did “PKCε activity,” at least as indirectly reflected in its membrane-to-cytosol ratio. However, one hepatic insulin-responsive metabolic pathway that was not “reversed” following the DS was DNL. Instead, activity in this pathway was significantly reduced in the DS group, presumably reflecting both the reduction in expression of key catalytic mediators of DNL (Fig. 4H) and reduced substrate supply. Another notable difference in the liver in the DS/CR mice compared to control and HFD-fed mice was the significant increase in ketones, which we suggest is a consequence of increased FA oxidation in the presence of reduced hepatic oxidative phosphorylation. At a whole-body level, this provides an alternative energy source for key tissues such as the brain in the context of reduced caloric intake.

WAT was very different insofar, as the reduction in glucose disposal after HFD feeding was not reversed in gWAT or iWAT after the DS, and the notable changes in lipolytic regulators such as ADRB3 expression and HSL activation were not reversed in either depot. The changes in glucose disposal correlated to some extent with changes in GLUT4 expression but the latter might simply be a consequence of altered insulin action rather than a causal mediator (*59*). We did not detect significant changes in the proximal insulin signaling components we evaluated. As WAT accounts for less than 10% of insulin-stimulated glucose disposal, its lack of reversal is perhaps not that unexpected in the context of restored glucose disposal at a whole-body level. Adipose DNL was very low in the obese HFD-fed mice and, in keeping with the liver data related to DNL, remained almost totally suppressed in the DS group.

Arguably, the most important metabolic function of adipose tissue relates to the regulation of lipolysis and postprandial lipid storage. In this context, fed and fasting NEFA levels were not elevated in the HFD-fed mice despite the increase in fat mass. However, whereas refeeding mice following an overnight fast results in a prompt increase in insulin and sharp fall in NEFA concentration in CD-fed control mice, obese HFD-fed mice manifest a marked blunting in this dynamic response to refeeding (fig. S9, G and H). This “adaptive” phenomenon has also been reported in humans (*60*) so we proceeded to explore its underlying molecular basis.

HSL primarily catalyzes the second step in intracellular TAG hydrolysis, with ATGL constituting the principal TAG lipase activity (*61*). However, HSL is activated by phosphorylation, which both activates the enzyme and facilitates its translocation to the surface of the lipid droplet where neutral lipid is stored (*62*), so immunoblotting for phosphorylated HSL should correspond with lipolytic activity. Plin1 is also phosphorylated by PKA and is then directly involved in the activation of ATGL via ABHD5 release (*63*). The consistent changes in Plin1 and HSL phosphorylation (Fig. 5G) therefore further attest to the suggestion that they can serve as readouts of intracellular lipolytic activation.

The signaling data in Fig. 5 suggest that WAT lipolytic activity is relatively high in CD-fed control mice after a 6-hour fast, and this activity is then very promptly suppressed when insulin rises. In HFD-induced obesity, lipolytic activity is substantially lower in each adipocyte and is then further reduced by insulin to a modest degree. As total fat mass is considerably higher in the obese mice, this results in very modest reduction in circulating NEFA levels.

The reduction in fasting lipolytic activity is likely to relate to the very significantly reduced ADRB3 expression observed in both gWAT and to a lesser extent in iWAT. Valentine *et al.* (*48*) suggested that this may relate to both homologous desensitization and to inflammatory mediators such as TNFα. Adipocyte death and the resulting inflammation are consistently more notable in gWAT than in iWAT in obese mice, so this could account for the greater reduction in ADRB3 in gWAT than in iWAT and for the fact that the reduction is greater after 18 weeks of HFD feeding (Fig. 7, F to H). In the DS group, the data indicate that the obesity-associated changes in lipolytic activity are not reversed in the 1- to 3-day time frame studied here and that the reduced fasting HSL phosphorylation is not simply related to higher insulin levels, as insulin concentrations in the DS mice are similar to those in the CD-fed controls. These findings suggest that the fact that changes in the regulation of WAT lipolysis are not reversed in the DS group plays a pivotal role in acutely restoring insulin sensitivity by contributing to lower circulating NEFA levels.

So, whereas the IR associated with HFD feeding–induced obesity is alleviated following DS/CR in terms of HOMA-IR and ITT, the changes noted in key insulin target tissues are not “simply reversed.” In WAT, lipolytic activity remains suppressed, and DNL is markedly suppressed, while glucose uptake is not restored. In skeletal muscle, ectopic lipid accumulation is alleviated to a large degree, and this likely reflects increased fat oxidation at least in part. Similarly, in the liver, ectopic fat accumulation is significantly reduced, but DNL is markedly reduced compared to the controls and HFD-fed mice, and ketogenesis is up-regulated, suggesting a catabolic response. The latter is in some ways similar to fasting but is different insofar, as NEFA levels are not elevated systemically as one would expect from fasting alone (*64*).

Overall, the calorie restriction appears to induce a rapid switch to a catabolic, rather than an insulin-stimulated anabolic state fueled by surplus energy intake. This is evidenced by a substantial increase in FA catabolic pathways in both muscle (Fig. 3, I and J) and liver (Fig. 4M), as well as an elevation in cellular respiration pathways in muscle tissue (fig. S4C) and lower plasma NEFA concentrations. Lipid synthesis was also reduced in the liver (Fig. 4G and fig. S7, F and I) and WAT (figs. S9, B and C, S10H, and fig. S11F). This is associated with a prompt and substantial fall in fed and fasting insulin concentrations and increase in glucose disposal, which we suggest is a consequence of the lower NEFA levels and thus reduced oxidative substrate competition. The above observations indicate that, while IR is reversed with DS, this results in a metabolically distinct state compared to CD, characterized by up-regulation of catabolic processes in the liver and muscle and lower circulating FA concentrations, thus ultimately improving insulin-stimulated glucose disposal.

HFD feeding is associated with surplus energy intake, which results in an anabolic state with higher insulin levels facilitating lipid storage primarily in adipocytes. This results in a significant increase in the size and number of adipocytes. If lipolysis continued in each of these adipocytes at the same rate as it had done before weight gain, then the lipolytic flux would inevitably increase substantially. Instead, what happens is that insulin levels increase promptly, suppressing lipolysis in each adipocyte. Furthermore, ADRB3 expression falls significantly, resulting in a sizable reduction in fasting state lipolytic activity and hence effectively restoring fasting lipolytic rates to a similar level to those of CD-fed controls.

However, this adipose tissue adaptation is associated with hyperinsulinaemia, which in itself is very likely to contribute to IR in both muscle and the liver. It also enhances lipid accumulation in these ectopic sites. Exactly how excess lipid accumulation in these tissues perturbs insulin action remains controversial, one prominent proposal being that DAG accumulation activates novel PKCs (ε in the liver and θ in muscle), which then impair insulin action by phosphorylating the insulin receptor itself (*15*, *65*) or immediate downstream signaling intermediates such as IRS1 (*57*, *66*). Our data from the liver are consistent with PKCε being involved in this way but the “assessment of PKCε activity” is indirect.

Adipose inflammation is often implicated in mediating obesity-associated IR. However, this is controversial, as it is primarily triggered by adipocyte death and could merely be a secondary phenomenon. We do see an increase in CLS by 8 weeks of HFD, more in the gWAT than in the iWAT, and this is more notable after 18 weeks of HFD feeding. These changes are not reversed following the DS, and yet whole-body insulin sensitivity is restored. Furthermore, TNFα expression does increase in the context of HFD feeding, and plasma TNFα was increased after 18 weeks on an HFD, but this was not reversed after the DS, arguing at least that in itself it is not sufficient to be the primary driver of IR. The data are, however, consistent with the increased adipose inflammation seen in male mice fed an HFD for 18 weeks, contributing to the incomplete alleviation of their IR following acute calorie restriction.

### Limitations

Human obesity is usually associated with sustained surplus energy intake, but this is not necessarily underpinned by a diet higher in fat, whereas in mice, conventional chow is ~65% carbohydrate, and switching to a high-fat formulation typically induces excess energy intake in C57BL/6 mice. These mice do then become “obese,” but their metabolic or physiological changes are also related to the much higher fat content in the diet. So called “Western diets,” which include a higher carbohydrate component, are also associated with weight gain but were not used in our study. We would anticipate similar changes but have not studied these to confirm this to date. In addition, the measurement of energy intake in our study was indirect and did not rely on the use of metabolic cages and bomb calorimetry.

Skeletal muscle accounts for most of insulin-stimulated glucose disposal. The radiolabeled ITT data herein indicated similar glucose disposal into skeletal muscle during the ITT in CD- and in HFD-fed mice, although this was in response to higher plasma insulin levels in the HFD-fed obese mice. In line with this data, the ITT AOC analyses suggest similar glucose clearance following insulin injection, although again this is in response to higher plasma insulin levels. Hyperinsulinaemic euglycaemic clamps are often used to document skeletal muscle IR but were not included in this study, as they typically involve surgery, which itself tends to result in reduced caloric intake for at least a few days. So further work will be needed to clarify change in skeletal muscle in this experimental paradigm. Several hypotheses have been put forward to explain obesity-associated IR in skeletal muscle (*14*, *67*–*71*), and we have to date not been able to support or refute these proposed mechanisms. In this context, we are also aware that many of insulin’s actions are mediated by changes in the phosphorylation of signaling intermediates not evaluated in our model.

## MATERIALS AND METHODS

### Experimental model and subject details

#### Mice

Both male and female wild-type C57BL/6J mice were purchased from Charles River (Charles River Ltd., Manston Rd., Margate, Kent, CT9 4LT) at 6 to 7 weeks of age. Mice were acclimatized for 1 week in the facility before commencing the study. Mice were maintained in ventilated cages with group housing (three to five per cage) on a 12-hour light/12-hour dark cycle (lights on 07:00 to 19:00), in a temperature-controlled (20° to 24°C) facility, with ad libitum access to water and diet. During the experimental protocol, all mice were either fed ad libitum (apart from the duration when mice underwent caloric restriction) or fasted before tests as stated.

All animal studies were regulated under the Animals (Scientific Procedures) Act 1986 Amendment Regulations 2012 following ethical review by the University of Cambridge Animal Welfare and Ethical Review Body under project licenses PP3183418 and PP6104469.

### Method details

#### Mouse study

Starting at the age of 8 weeks, mice were fed either a standard rodent CD (SAFE Diets 105; 22.4% kcal protein, 13.3% kcal fat and 64.3% kcal carbohydrate) or an HFD with 60% kcal fat (D12492; Research Diets, New Brunswick, NJ, USA; 20% kcal protein and 20% kcal carbohydrate) for the duration of the study (8 or 18 weeks). For a subgroup of HFD-fed mice, after 8 or 18 weeks of feeding, diet was switched from HFD to CD for 1 or 3 days or restricted by 70% calories on HFD for 18 hours. For all cohorts, body weight and cage food intake were recorded weekly before DS or calorie restriction and daily afterward. Body composition was determined at the end of the study using a magnetic-resonance whole-body composition analyzer (EchoMRI, Houston, TX, 77079). Blood samples were collected either from lateral tail vein into heparinized micro blood tubes (Hawksley, 01605-00) or by cardiac puncture, centrifuged at 13,000*g* for 4 min, and plasma was collected for subsequent analyses.

At the end of the study, mice were euthanized by cervical dislocation in most cohorts. In the CR (calorie restriction) cohort, the physiological lipolysis cohort and the phosphate-buffered saline (PBS) versus insulin treatment cohort, mice were euthanized by CO_2_ overdose. Tissues were harvested, weighed, and frozen at –80°C or fixed in 10% neutral buffered formalin (VWR Chemicals, 9713.5000) for subsequent analyses.

#### Food intake

Cage food intake was calculated by measuring the food in the hopper at the start and at the end of the week or day. Furthermore, bedding was sieved to remove any unconsumed food particles, which were weighed and subtracted from the total food intake measurement. Food intake per mouse was calculated accordingly by dividing cage food intake by the number of mice.

#### Plasma assays

Mouse plasma sample measurements were performed by the National Institute for Health Research (NIHR) Cambridge BRC Core Biochemical Assay Laboratory. Mouse leptin and insulin were measured using a single-plex or duplex Meso Scale Discovery assay kit (Rockville, MD, USA, K152BYC-2 for leptin and K152BZC-3 for insulin). TAG was measured using an enzymatic assay (Siemens Healthcare, DF69A). Glucose was measured using the hexokinase-glucose-6-phosphate dehydrogenase method (Siemens Healthcare, DF30). NEFA was measured using a Roche Free Fatty Acid Kit (Sigma-Aldrich, 11383175001). Inflammatory cytokines were measured using MSD Proinflammatory Panel 1 (Rockville, MD, product code K15048D-2). All assays were performed according to the manufacturer’s instructions.

#### Insulin tolerance test

Mice were fasted for 6 hours before intraperitoneal administration of insulin (0.75 to 1 U/kg of body weight for male mice and 0.5 to 0.75 U/kg of body weight for female mice) in normal saline. Blood glucose was measured before insulin injection and at 10, 20, 30, 60, 90, and 120 min after injection.

#### HOMA-IR and QUICKI calculation

HOMA-IR was calculated as fasting glucose (mM) × fasting insulin (μU/ml)/22.5. QUICKI was calculated as 1/[log_10_(fasting insulin μU/ml) + log_10_(fasting glucose mg/dl)].

#### ITT under anesthesia

ITT under anesthesia was performed according to a previously published protocol (*34*). After 6 hours of fasting, mice were injected with 80 mg/kg of body weight pentobarbitone intraperitoneally. Mice were then secured on a heat pad after good quality of anesthesia was confirmed using toe-pinch reflex. Abdominal cavity was opened, and insulin at 0.75 U/kg calculated on the basis of the average body weight of CD-fed mice was injected together with 7.5 mCi [^3^H]2-DOG tracer into the hepatic portal vein. Blood glucose was measured, and blood was sampled from tail vein before injection and at 2, 5, 10, 15, 20, and 30 min postinjection. After 30 min, mice were terminated by cervical dislocation, and tissues including soleus, gastrocnemius, quadriceps, gWAT, and iWAT were excised for subsequent analyses.

Blood samples were deproteinized with ZnSO_4_ and Ba(OH)_2_, followed by centrifugation at ×1000*g* for 5 min. The supernatant was transferred into a scintillation vial with 3 ml of scintillant added. ^3^H disintegrations per minute (dpm) was counted using a scintillation counter.

Tissues were powdered in liquid nitrogen using a mortar and pestle. Around 40 mg of tissue was homogenized in ddH_2_O by sonication before centrifugation at ×13,000*g* for 15 min. Supernatant was transferred to a new tube, diluted with ddH_2_O, and added into a chromatography column containing AG 1-X8 resin. Columns were washed three times with ddH_2_O and eluted with elution buffer [1% trifluoroacetic acid (Sigma-Aldrich, 302031) and 2 M NaCl (Sigma-Aldrich, S7653)] into scintillation vials. After adding scintillation fluid, samples were vortexed, and ^3^H dpm was measured using a scintillation counter to quantify [^3^H]2-DOG-6-P.

#### Insulin signaling assessment

Mice were fasted for 6 hours before intraperitoneal injection of either PBS or insulin (1 U/kg based on the average body weight of CD-fed mice). Mice were euthanized 20 min postinjection, and tissues were harvested and frozen at –80°C for subsequent analyses. Plasma was sampled pre- and postinjection via tail vein and posteuthanasia via cardiac puncture.

#### Lipolysis assessment

For the cohorts where we assessed insulin-regulated lipolysis, all mice were fasted overnight for 12 hours, followed by refeeding for 2 hours with CD. Blood samples were collected both after overnight fasting and after refeeding for the measurement of insulin, glucose, and NEFA levels.

#### Western blot

Frozen tissue was crushed in liquid nitrogen using a pestle and mortar. Powdered tissue was lysed in radioimmunoprecipitation assay buffer (Sigma-Aldrich, R0278) containing protease inhibitor (Roche, 18836170001) and phosphatase inhibitor (Roche, 04906845001). Samples were incubated on a shaker at 4°C for 30 min, followed by centrifugation at ×13,000*g* for 15 min at 4°C to remove lipids and insolubilized tissue. Supernatant was collected, and protein concentration was quantified using Bio-Rad DC Protein Assay Kit (5000113, 5000114, and 5000115). Protein lysates were mixed with 4× NuPAGE LDS buffer (Thermo Fisher Scientific, NP0007) containing dithiothreitol (final concentration of 50 mM) and denatured for 10 min at 65°C (note: samples were not heated for ADRB3 immunoblotting). Proteins from tissue lysates were resolved on Invitrogen NuPAGE 4–12% Bis-Tris gels and transferred onto a nitrocellulose membrane using an invitrogen iBlot2 machine. Membranes were washed in tris-buffered saline with 0.1% (v/v) Tween 20 (Sigma-Aldrich, 9005-64-5) (TBST) before blocking in 5% (w/v) skimmed milk in TBST for 1 hour at room temperature (RT). Membranes were incubated with primary antibodies at 4°C overnight, followed by washes with TBST and incubation with horseradish peroxidase (HRP)–conjugated secondary antibodies for 1 hour at RT. After three washes with TBST, blots were developed using Immobilon Western HRP Substrate Peroxide Solution and Immobilon HRP Substrate Luminol Reagent (Millipore, WBKLS0500), and images were acquired on Bio-Rad ChemiDoc Imaging system. Primary antibodies used in this study include the following: InsRβ [Santa Cruz (SC), 57342], pInsRβ Tyr1162 [Cell Signaling Technology (CST), 3918], Akt (CST, 2920), pAkt Ser473 (CST, 4060), pAkt Thr308 (CST, 13038), pAS160 Thr642 (CST, 8881), GLUT4 (made in house), pGSK3 Ser9/21 (CST, 9331), calnexin [Abcam (Ab), 22595], tPlin1 (Vala Sciences, 4854), PKCε (BD Biosciences, 610085), Na^+^- and K^+^-dependent ATPase (Na^+^,K^+^-ATPase) (Ab, 7671), glyceraldehyde-3-phosphate dehydrogenase (GAPDH) [GeneTex (GTX), 100118], FAS (Ab, 22759), ACLY (Proteintech, 15421-1-AP), SCD1(CST, 2794), HSL (CST, 4107), pHSL Ser660 (CST, 45804), pPlin1 Ser522 (Vala Sciences, 4856), ABHD5 (Abnova, H00051099-M01), ATGL (CST, 2138), leptin (Ab, 9749), adiponectin (Ab, 85827), and ADRB3 (Ab, 94506). Western blots were quantified using the ImageJ software using Integrated Density.

#### RNA preparation and quantitative polymerase chain reaction

Frozen tissue was crushed in liquid nitrogen using a pestle and mortar. Approximately 30 to 50 mg of powdered tissue was lysed in 800 μl of TRI Reagent (Sigma-Aldrich), followed by the addition of 200 μl of chloroform (Sigma-Aldrich). Samples were vortexed and centrifuged at ×13,000*g* for 15 min at 4°C. Clear upper phase was then transferred into a ribonuclease-free tube and mixed with an equal volume of 70% ethanol before loading onto RNA isolation spin columns (QIAGEN RNeasy Mini Kit 250). RNA was then extracted according to the manufacturer’s instructions.

RNA concentration and quality were determined using a NanoDrop Microvolume Spectrophotometer. Total RNA of 400 ng was converted to cDNA using LunaScript (New England Biolabs) following DNase digestion using RQ1 DNase (Promega). Quantitative reverse transcription polymerase chain reaction (RT-PCR) was performed with TaqMan Universal PCR Mix or SYBR Green PCR Master Mix on the QuantStudio 5 Flex Real-Time PCR system (Thermo Fisher Scientific). All reactions were performed in duplicate, and *C*_t_ values were obtained. Relative differences in gene expression were normalized to housekeeping genes B2m and 36b4 using geometric mean. Primer sequences are the following: mouse Adrb3, GGCCCTCTCTAGTTCCCAG (forward); mouse Adrb3, TAGCCATCAAACCTGTTGAGC (reverse); mouse Fgf21, AGATCAGGGAGGATGGAAC (forward); mouse Fgf21, TCAAAGTGAGGCGATCCATA (reverse); mouse F4/80, CAGATACAGCAATGCCAAGCA (forward); mouse F4/80, GATTGTGAAGGTAGCATTCACAAGTG (reverse); mouse CD68, GA-TTGTGAAGGTAGCATTCACAAGTG (forward); mouse CD68, GATGAATTCTGCGCCATGAA (reverse); mouse monocyte chemoattractant protein-1 (MCP1), CCACTCACCTGCTGCTACTCA (forward); mouse MCP1, TGGTGATCCTCTTGTAGCTCTCC (reverse); mouse TNFα, CATCTTCTCAAAATTCGAGTGACAA (forward); mouse TNFα, TGGGAGTAGACAAGGTACAACCC (reverse); mouse macrophage galactose N-acetyl-galactosamine specific lectin 2 (MGL2), GGAGTCTCCAAAGTTTGCTCTAA (forward); mouse MGL2, AGGTGGGTCCAAGAGAGGAT (reverse); mouse CD206, GCATGGGTTTTACTGCTACTTGATT (forward); mouse CD206, C-AGGAATGCTTGTTCATATCTGTCTT (reverse); mouse CD209, GCCCTCTGGATGAGGAACTG (forward); mouse CD209, GGCACCCTGTGAAGCTACTGA (reverse); mouse 36b4, AGATGCAGCAGATCCGCAT (forward); mouse 36b4, GTTCTTGCCCATC-AGCACC (reverse); mouse B2m, ACTGATACATACGCCTGCAGAGTT (forward); mouse B2m, TCACATGTCTCGATCCCAGTAGA (reverse).

#### PKCε translocation assay

PKCε translocation assay was performed according to a previously published protocol (*13*). Around 50 mg of liver tissue was homogenized in ice-cold buffer A [20 mM tris-HCl (pH 7.4), 1 mM EDTA, 0.25 mM EGTA, and 250 mM sucrose], with freshly added protease inhibitor (Roche, 18836170001) and phosphatase inhibitor (Roche, 04906845001). Samples were then centrifuged at ×300*g* at 4°C for 5 min to remove debris. An aliquot of the supernatant was saved as the whole tissue lysate, the rest was transferred into a new tube and centrifuged at ×100,000*g* at 4°C for 60 min, and an aliquot of the supernatant was saved as the cytosolic fraction. The pellet was washed once in ice-cold buffer B [250 mM tris-HCl (pH 7.4), 1 mM EDTA, 0.25 mM EGTA, and 2% Triton X-100], with freshly added protease and phosphatase inhibitors, and resuspended in buffer B by sonication at 20% power for 10 s using a probe sonicator. Samples were incubated at 4°C for 45 min to solubilize membrane proteins and centrifuged at ×100,000*g* at 4°C for 60 min. An aliquot of the supernatant was saved as the membrane fraction. The resulting protein samples were subjected to Western blot analysis as described above. The ratio between membrane PKCε (normalized to Na^+^,K^+^- ATPase intensity) to cytosolic PKCε (normalized to GAPDH intensity) was calculated as an index of PKCε translocation.

#### DNL measurements

Deuterium oxide was used in this study for the in vivo tracing of DNL. Mice received an intraperitoneal injection of deuterium oxide (D_2_O; 20 μl/g; Sigma-Aldrich, St. Louis, MO, USA), and D_2_O was added to their drinking water to 4% final concentration, provided ad libitum. Intraperitoneal injections and water supplementation occurred 24 hours before culling.

Protein precipitation extraction method (*72*): Glass pipettes were used throughout the procedure. Extraction solution was prepared with 2:1 (v/v) high-performance liquid chromatography (HPLC) grade chloroform and methanol containing glyceryl tritridecanoate (250 mg/ml; Sigma-Aldrich) and 1,2-dipentadecanoyl-*sn*-glycero-3-phosphocholine (125 mg/ml; Avanti Polar Lipids, 850350P). Approximately 20 mg of tissue per sample was added to 0.6 ml of extraction solution. Tissues were homogenized for 1 min at 1 m/s in homogenizer tube with ceramic beads (Lysing Matrix D, MP Biomedicals, 1169130-CF). A total of 0.4 ml of extraction solution was added, and samples were vortexed for a further 1 min. Following this, 0.2 ml of acetone was added, and samples were vortexed for another 1 min. Vortexed samples were centrifuged at ×1400*g* for 15 min at RT. The supernatant was transferred to a glass vial and dried under a stream of nitrogen. Dried lipids were stored overnight at −20°C.

Derivatization: Isolated lipids were derivatized to FA methyl esters (FAMEs) by adding a mixture of 375 μl of chloroform, 375 μl of methanol, and 125 μl of 10% boron trifluoride (Sigma-Aldrich, 15716) to each sample, vortexing thoroughly, and incubating at 80°C for 1.5 hours. After cooling at RT for 30 min, 500 μl of MilliQ water and 1 ml of hexane were then added, and each sample vortexed before centrifuging at ×1400*g* for 5 min. The upper layer was transferred to Autosampler vials for subsequent gas chromatography–mass spectrometry (GC-MS). Samples were stored at −20°C until GC-MS could be performed.

GC-MS was performed using a 7890B gas chromatography system coupled to a 5977A mass spectrometer and an AS3000 autosampler (Agilent). Separation was achieved on a TR-FAME column (Thermo Fisher Scientific, 260M142P) with helium as the carrier gas at a constant flow rate of 1.5 ml/min. The inlet temperature was set to 230°C, and 1 μl of each sample was injected. The oven temperature program was as follows: an initial hold at 100°C for 2 min; ramp at 25°C/min to 150°C; ramp at 2.5°C/min to 162°C and hold for 3.8 min; ramp at 4.5°C/min to 173°C and hold for 5 min; ramp at 5°C/min to 210°C; and then ramp at 40°C/min to 230°C and hold for 0.5 min. Samples were first analyzed in scan mode [45 to 400 mass/charge ratio (*m/z*)], followed by single ion monitoring mode for *m/z* of 270 to 275 to assess palmitate mass isotopomer distribution.

Identification of specific FAME peaks was based on retention time and mass spectra compared against a standard curve generated with a Food Industry FAME mix (Restek, Bellefonte, PA, USA, 35077). Samples in which FAME peak heights exceeded 1 × 10^8^ U were diluted in hexane and reinjected. Peak integration and quantification were performed with MassHunter Workstation Quantitative Analysis software (version B.07.00, Agilent). High-abundance ions from the total ion chromatogram were selected for calculation of each FA peak. Peak area values for each FA were normalized to the peak area values of the Food Industry FAME mix standard curve for quantification. DNL rates were determined on the basis of the calculated mass isotopomer distribution of palmitate as described by Bidault *et al.* (*73*).

#### Histology

Tissues were dissected and placed into 10% neutral buffered formalin for 48 hours at RT before being transferred to 70% ethanol and stored at 4°C. After fixation, tissues were infiltrated with paraffin wax using a VIP6 tissue processor (Sakura Finetek, Tokyo, Japan) and embedded into paraffin blocks using the Histostar Embedding Station (Epredia, Portsmouth, USA) before being trimmed and sliced into 4-μm sections using a Leica RM2235 rotary microtome (Leica Biosystems, Nussloch, Germany) and mounted onto Superfrost Plus slides (Epredia, Portsmouth, USA). Tissues were then stained either using haemotoxylin (Pioneer Research Chemicals, Colchester, UK, PRC/R/42) and eosin (Pioneer Research Chemicals, Colchester, UK, PRC/66/1) or F4/80 (1:20, Bio-Rad, Hercules, USA, MCA497). Following staining, samples were mounted using Pertex mounting medium (Pioneer Research Chemicals, Colchester, UK, PRC/R/750) and 22 × 40 mm coverslips (VWR, Radnor, USA, 631-0136) and left to dry overnight at RT before cleaning and imaging.

Slides were imaged under bright field using the Axio Scan.Z1 Image Acquisition system (Zeiss, Oberkochen, Germany). Histological analyses of these images were performed using the HALO Image Analysis software (Indica Labs, Albuquerque, NM, USA).

#### Adipocyte size quantification

Adipocyte size was quantified manually using the ImageJ software (Rasband W., National Institutes of Health, Bethesda, MD, USA). Screen captures were taken from the bright-field images captured using the HALO image analysis software. Scale was set manually using the original HALO image scaling, and cells were circled by hand to measure total area in square micrometers. A minimum of 15 adipocytes per mouse was measured.

#### Lipidomics

The protein precipitation liquid extraction protocol was described previously (*72*). Briefly, to each sample (~30 mg of muscle tissue or ~35 mg of liver tissue), we added 400 μl of chloroform to each sample, along with a single 5-mm stainless steel ball bearing. The samples were then homogenized using a Bioprep 24-1004 homogenizer (Allsheng, Hangzhou, China) run at a speed of 4.5 m/s and a time of 30 s for 2 cycles. Following this, 250 μl of chloroform, 250 μl of methanol, 100 μl of the LIPID+CARNITINE internal standard (1 to 10 μM in methanol), and 400 μl of acetone were added to each sample. The samples were vortexed thoroughly. The samples were then centrifuged for 10 min at ~×20,000*g* to pellet any insoluble material. The supernatant was pipetted into separate 2-ml screw cap amber-glass autosampler vials (Agilent Technologies, Cheadle, United Kingdom). The extracts were dried down to dryness using a Concentrator Plus system (Eppendorf, Stevenage, United Kingdom) run for 60 min at 60°C.

For lipidomics liquid chromatography–mass spectrometry (LC-MS_ analysis, the samples were reconstituted in 100 μl of 2:1:1 (propan-2-ol, acetonitrile, and water, respectively) and then thoroughly vortexed. The reconstituted sample was transferred into a 250-μl low-volume vial insert inside a 2-ml amber glass autosample vial ready for LC-MS lipidomics analysis. Full chromatographic separation of intact lipids was achieved using Waters Acquity H-Class HPLC System (Waters, Hertfordshire, United Kingdom) with the injection of 10 μl onto a Waters Acquity Premier UPLC CSH C18 column (1.7 μm, inside diameter (ID) of 2.1 mm by 50 mm, and maintained at 55°C). Mobile phase A was 6:4 of acetonitrile and water with 10 mM ammonium formate. Mobile phase B was 9:1 of propan-2-ol and acetonitrile with 10 mM ammonium formate. The flow was maintained at 500 μl/min through the following gradient: 0.00 min_40% mobile phase B; 1.5 min_40% mobile phase B; 8.00 min_99% mobile phase B; 10.00 min_99% mobile phase B; 10.10 min_40% mobile phase B; 12.00 min_40% mobile phase B. The sample injection needle was washed using 9:1 of propan-2-ol and acetonitrile (strong wash) and 2:1:1 of propan-2-ol, acetonitrile, and water (weak wash). The mass spectrometer used was the Thermo Fisher Scientific Q-Exactive Orbitrap with a heated electrospray ionization source (Thermo Fisher Scientific, Hemel Hempstead, United Kingdom). The mass spectrometer was calibrated immediately before sample analysis using positive and negative ionization calibration solution (recommended by Thermo Fisher Scientific). In addition, the mass spectrometer scan rate was set at 4 Hz, giving a resolution of 35,000 (at 200 *m/z*) with a full-scan range of *m/z* 100 to 1800 with continuous switching between positive and negative mode. The data processing involved the integration of the extracted ion chromatogram peaks for each target lipid species (~1600 species) and the stable isotope–labeled internal standards at their expected retention time. The area ratio response of the target lipid to the corresponding internal standard was converted into nanomoles results normalized to the amount of tissue extracted (nanomoles per milligram). All results were subjected to blank correction and comprehensive quality checking before further statistical analysis.

#### Ketone body measurement

To each sample (10 μl of plasma), we added 100 μl of methanol containing the AcAc-13C_4_ internal (at 5 μM). The samples were vortexed thoroughly and then centrifuged for 10 min at ~×20,000*g* to pellet any insoluble material. The supernatant was pipetted into a 250-μl low-volume vial insert inside 2-ml screw cap amber-glass autosampler vials (Agilent Technologies, Cheadle, United Kingdom) ready for LC-MS hydroxybutyrate (α-hydroxybutyrate, β-hydroxybutyrate, β-hydroxyisobutyrate, γ-hydroxybutyrate, and AcAc) analysis.

Full chromatographic separation of hydroxybutyrates was achieved using Waters Acquity H-Class HPLC System (Waters, Hertfordshire, United Kingdom) with the injection of 2 μl onto a Imtakt Scherzo SM-C18 mixed-mode (reversed phase, weak anion exchange and weak cation exchange: 150 mm–by–3 mm internal diameter, 3-μm particle size) maintained at 30°C. Mobile phase A was water with 0.3% formic acid. Mobile phase B was acetonitrile with 1% formic acid. The flow was maintained at 500 μl/min through the following gradient: 0.00 min_0% mobile phase B; 2.00 min_0% mobile phase B; 5.00 min_10% mobile phase B; 5.10 min_50% mobile phase B; 7.00 min_50% mobile phase B; 7.10 min_0% mobile phase B; 10.00 min_0% mobile phase B. The sample injection needle was washed using 1:1 of water:methanol with 0.1% acetic acid (strong wash) and water (weak wash). The mass spectrometer used was the Thermo Fisher Scientific Q-Exactive Orbitrap with a heated electrospray ionization source (Thermo Fisher Scientific, Hemel Hempstead, United Kingdom). The mass spectrometer was calibrated immediately before sample analysis using positive and negative ionization calibration solution (recommended by Thermo Fisher Scientific). In addition, the mass spectrometer scan rate was set at 2 Hz, giving a resolution of 70,000 (at 200 *m/z*) with a full-scan range of *m/z* 100 to 110 in negative mode and *m/z* 75 to 150 in positive mode. The data processing involved the integration of the extracted ion chromatogram peaks for each target hydroxybutyrate species (α-hydroxybutyrate, β-hydroxybutyrate, β-hydroxyisobutyrate, γ-hydroxybutyrate, and AcAc) and the stable isotope–labeled AcAc-13C_4_ internal standards at their expected retention time. The area ratio response of the target ion to the corresponding internal standard was converted into micromolar results. All results were subjected to blank correction and comprehensive quality checking before further statistical analysis.

#### Proteomics studies

Tandem mass tag (TMT) labeling: TMT labeling was performed according to the manufacturer’s protocol (www.thermofisher.com/order/catalog/product/A44522#/A44522). A total of 100 mg of each digested protein sample was labeled individually with 16 TMTpro tags. Postlabeling, samples were combined and cleaned on Sep-Pak C18 cartridge, dried, and dissolved in 20 mM ammonium formate (pH 10). The solution was then pipetted into a sample vial and placed into the autosampler of a Waters Acquity UPLC pump (Waters Corporation, Milford, MA).

High-pH first dimension reverse-phase fractionation: The following LC conditions were used for the fractionation of the TMT samples: Desalted peptides were resuspended in 0.1 ml of 20 mM ammonium formate (pH 10.0) + 4% (v/v) acetonitrile. Peptides were loaded onto an Acquity bridged ethyl hybrid C18 UPLC column (Waters; ID of 2.1 mm by 150 mm, 1.7-μm particle size) and profiled with a linear gradient of 5 to 60% acetonitrile +20 mM ammonium formate (pH 10.0) over 60 min at a flow rate of 0.25 ml/min. Chromatographic performance was monitored by sampling eluate with a diode array detector (Acquity UPLC, Waters) scanning between wavelengths of 200 and 400 nm. Samples were collected in 1-min increments and reduced to dryness by vacuum centrifugation.

LC–tandem MS (MS/MS): Dried fractions from the high-pH reverse-phase separations were resuspended in 30 ml of 0.1% formic acid and placed into a glass vial. A total of 1 ml of each fraction was injected by the HPLC autosampler and separated by the LC method detailed below. Thirty fractions were combined into pairs (i.e., the first fraction with the middle fraction, etc., giving a total of 15) and were analyzed by LC-MS/MS.

LC-MS/MS experiments were performed using a Dionex Ultimate 3000 RSLCnano UPLC (Thermo Fisher Scientific Inc., Waltham, MA, USA) system and a Lumos Orbitrap mass spectrometer (Thermo Fisher Scientific Inc., Waltham, MA, USA). Peptides were loaded onto a precolumn (Thermo Fisher Scientific PepMap 100 C18; 5-mm particle size, 100-Å pore size, 300-mm ID × 5-mm length) from the Ultimate 3000 autosampler with 0.1% formic acid for 3 min at a flow rate of 15 ml/min. After this period, the column valve was switched to allow elution of peptides from the precolumn onto the analytical column. Separation of peptides was performed by C18 reverse-phase chromatography at a flow rate of 300 nl/min using a Thermo Fisher Scientific reverse-phase nano EASY-Spray column (Thermo Fisher Scientific PepMap C18; 2-mm particle size, 100-Å pore size, 75-mm ID × 50-cm length). Solvent A was water + 0.1% formic acid, and solvent B was 80% acetonitrile and 20% water + 0.1% formic acid. The linear gradient used was 2 to 40% B in 105 min (total LC run time was 120 min including a high organic wash step and column reequilibration).

The eluted peptides from the C18 column LC eluant were sprayed into the mass spectrometer by means of an EASY-Spray source (Thermo Fisher Scientific Inc.). All *m/z* values of eluting peptide ions were measured in an Orbitrap mass analyzer, set at a resolution of 120,000, and were scanned between *m/z* of 380 and 1500 kDa. Data-dependent MS/MS scans (Top Speed) were used to automatically isolate and fragment precursor ions by collision-induced dissociation [normalized collision energy (NCE): 35%], which were analyzed in the linear ion trap. Singly charged ions and ions with unassigned charge states were excluded from being selected for MS/MS, and a dynamic exclusion window of 70 s was used. The top 10 most abundant fragment ions from each MS/MS event were then selected for a further stage of fragmentation by Synchronous Precursor Selection MS3 (*74*) in the higher-energy collisional dissociation cell using an NCE of 55%. The *m/z* values and relative abundances of each reporter ion and all fragments (mass range from 100 to 500 kDa) in each MS3 step were measured in the Orbitrap analyzer, which was set at a resolution of 50,000.

### Quantification and statistical analysis

#### Statistical analyses

Quantitative data are reported as mean ± SEM. As indicated in the figure legends, differences between means were assessed by one-way analysis of variance (ANOVA) or two-way ANOVA with correction for multiple comparisons using the GraphPad Prism software (GraphPad, San Diego). Statistical significance was defined as *P* < 0.05.

#### Proteomics analyses

Proteome Discoverer v2.1 (Thermo Fisher Scientific) and Mascot (Matrix Science) v2.6 were used to process raw data files. Data were aligned with the UniProt *Mus musculus* database (63,425 sequences; 28,187,043 residues) and a common repository of adventitious proteins (cRAP) v1.0. Protein identification allowed an MS tolerance of ±10 parts per million and an MS/MS tolerance of ±0.8 kDa along with permission of up to two missed tryptic cleavages. Quantification was achieved by calculating the sum of centroided reporter ions within a ±2–millimass unit window around the expected *m/z* for each of the four TMT reporter ions.

All comparative analyses were performed with the R statistical language. The R package QFeatures was used for processing proteomics data (*75*). Briefly, this entailed missing value removal (instances where a protein identified but not quantified in all channels was rejected from further analysis) and log_2_ transformation of the raw data, followed by sample normalization using a center median normalization approach in QFeature (this translates all sample columns so that they all match the grand median). Hierarchical cluster analyses were performed using function “hclust.” Protein differential abundance was evaluated using the limma package (*76*). Differences in protein abundances were statistically determined using an empirical Bayes-moderated linear model. *P* values were adjusted for multiple testing by the Benjamini-Hochberg method (*77*).

#### Lipidomics analyses

Lipidomics data analyses were performed with the R statistical language, following published workflow using QFeature package (*75*). Briefly, this entailed missing value removal (instances where a lipid species identified but not quantified in all channels was rejected from further analysis) and log_2_ transformation of the raw data. Hierarchical cluster analyses were performed using function hclust. Lipid differential concentration was evaluated using the Limma package (*76*). Differences in lipid abundances were statistically determined using an empirical Bayes-moderated linear model. *P* values were adjusted for multiple testing by the Benjamini-Hochberg method (*77*).
