# Supplementary material for: Integrated analysis of insulin resistance reveals metabolic remodeling following diet switch–triggered calorie reduction
Source: Sci Adv. 2026 May 6;12(19):eaed0535. doi: 10.1126/sciadv.aed0535 (PMC13148310; doi:10.1126/sciadv.aed0535)
Supplement: Supplementary file 1 — Figs. S1 to S12 [file sciadv.aed0535_sm.pdf]

Science Advances
AAAS

Supplementary Materials for

Integrated analysis of insulin resistance reveals metabolic remodeling following diet switch–triggered calorie reduction

Xiaowen Duan *et al.*

Corresponding author: Xiaowen Duan, xd253@cam.ac.uk; David B. Savage, dbs23@cam.ac.uk

Sci. Adv. **12**, eaed0535 (2026)
DOI: 10.1126/sciadv.aed0535

This PDF file includes:

Figs. S1 to S12

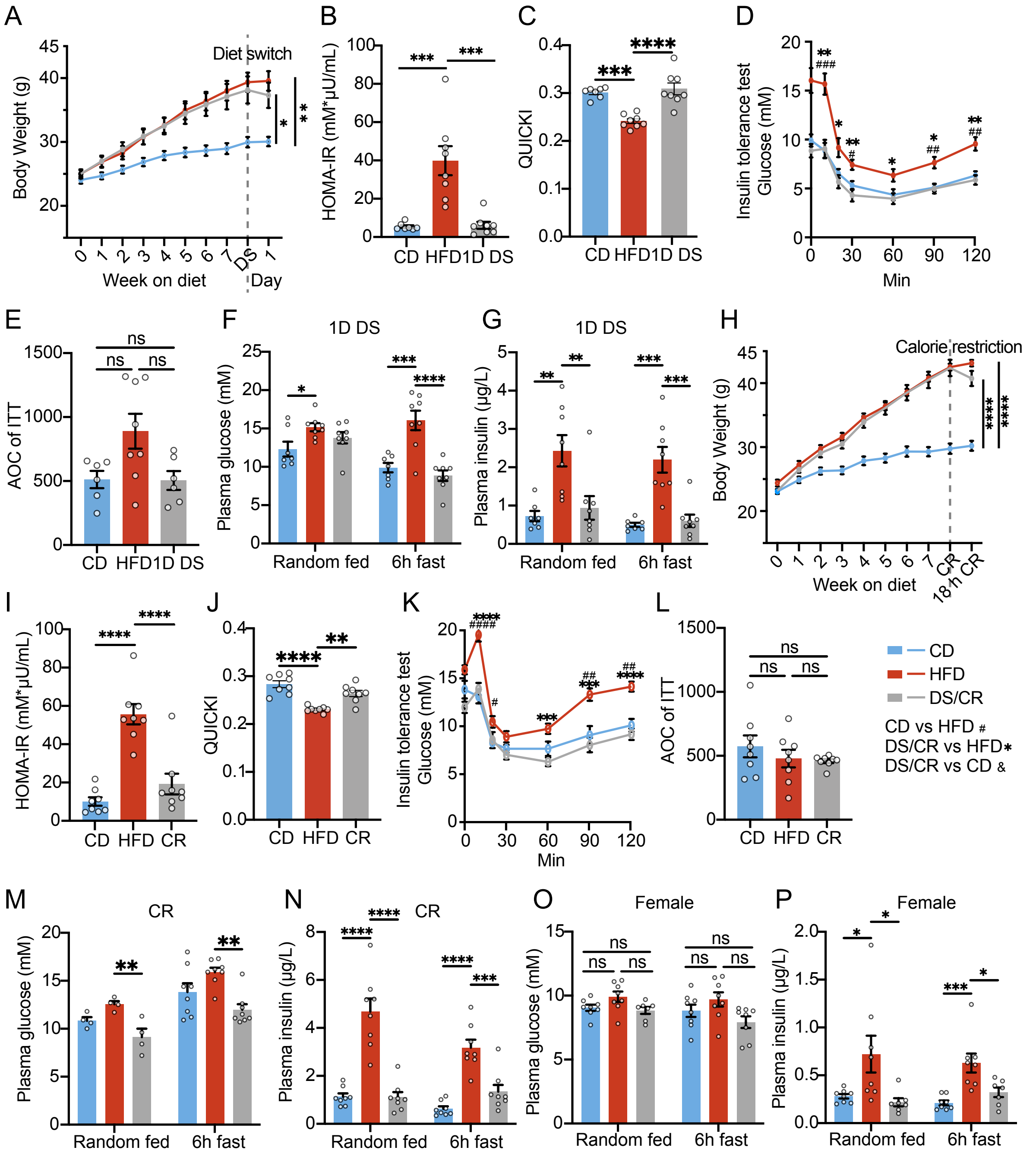

Figure S1. Parameters of mice from different calorie-restriction models, related to Figure 1. (**A-G**) Body weight (**A**), HOMA-IR (**B**), QUICKI (**C**), ITT (**D**), AOC of ITT (**E**), plasma glucose (**F**), insulin (**G**) of male mice fed with CD, HFD or those after one day DS from HFD to CD. Insulin was administered at a dose of 1 U/Kg body weight for the ITT. (**H-N**) Body weight (**H**), HOMA-IR (**I**), QUICKI (**J**), ITT (**K**), AOC of ITT (**L**), plasma glucose (**M**), insulin (**N**) of male mice fed with CD, HFD, or those after CR by 70% on HFD for 18 hours. Insulin was administered at a dose of 0.75 U/Kg body weight for the ITT. (**O-P**) Plasma glucose (**O**) and insulin (**P**) of female mice fed with CD, HFD or those after three days DS.

Error bars represent mean ± SEM. Significance for (**A**), (**H**) was calculated based on the body weight at the end of the study. Significance for (**A-C**), (**E-G**), (**H-J**), (**L-P**) was determined utilizing Ordinary one-way ANOVA

with Tukey test to correct for multiple comparisons. Significance for (**D**), (**K**) was calculated for each time point, utilizing Mixed-effects analysis with Tukey correction for multiple comparisons, # indicates the comparison between CD and HFD groups, * is for the comparison between DS/CR and HFD groups, and & is for the comparison between DS/CR and CD groups. * $p < 0.05$, ** $p < 0.01$, *** $p<0.001$, and **** $p < 0.0001$. (Abbreviations: CD, chow diet; HFD, high fat diet; DS, diet switch; CR, calorie restriction; HOMA-IR, homeostatic model assessment for insulin resistance; QUICKI: quantitative insulin sensitivity check index; ITT, insulin tolerance test; AOC, area of the curve)

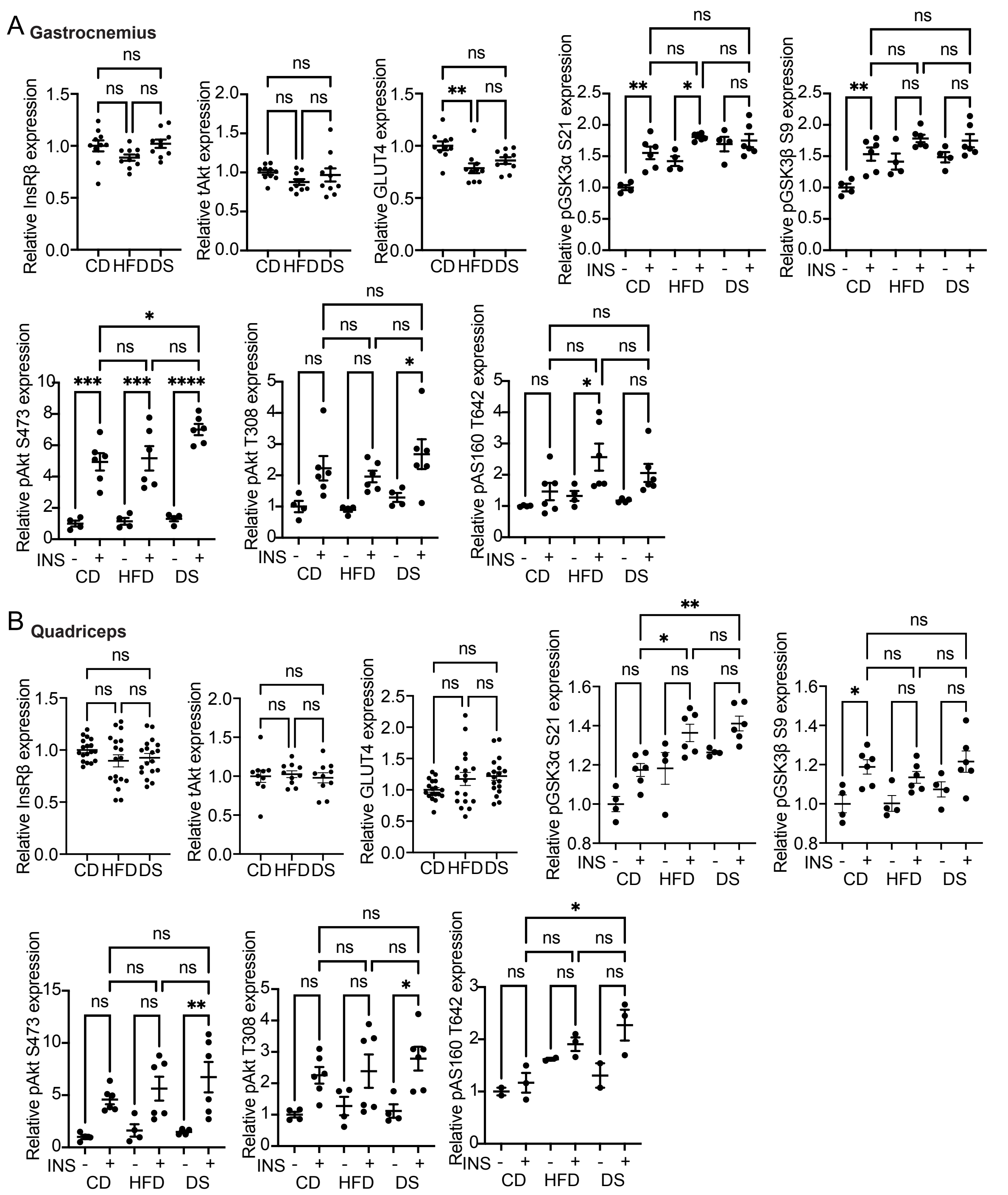

Figure S2. Quantification of western blot data from muscles of male mice, related to Figure 3. (**A**) InsRβ, Akt, GLUT4, pGSK3α S21, pGSK3β S9, pAkt S473, pAkt T308, and pAS160 T642 expression relative to loading control in gastrocnemius muscle from CD, HFD fed male mice or those subject to three days DS from HFD to CD (with PBS or insulin injection for protein phosphorylation). (**B**) InsRβ, Akt, GLUT4, pGSK3α S21, pGSK3β S9, pAkt S473, pAkt T308, and pAS160 T642 expression relative to loading control in quadriceps muscle from CD, HFD fed male mice or those subject to three days DS from HFD to CD, with PBS or insulin injection. Insulin was administered at a dose of 1 U/Kg of the average body weight of CD group mice.

Error bars represent mean ± SEM. Significance was determined using Ordinary one-way ANOVA with Sidak or Tukey test to correct for multiple comparisons. * $p < 0.05$, ** $p < 0.01$, *** $p<0.001$, and **** $p < 0.0001$. (Abbreviations: CD, chow diet; HFD, high fat diet; DS, diet switch)

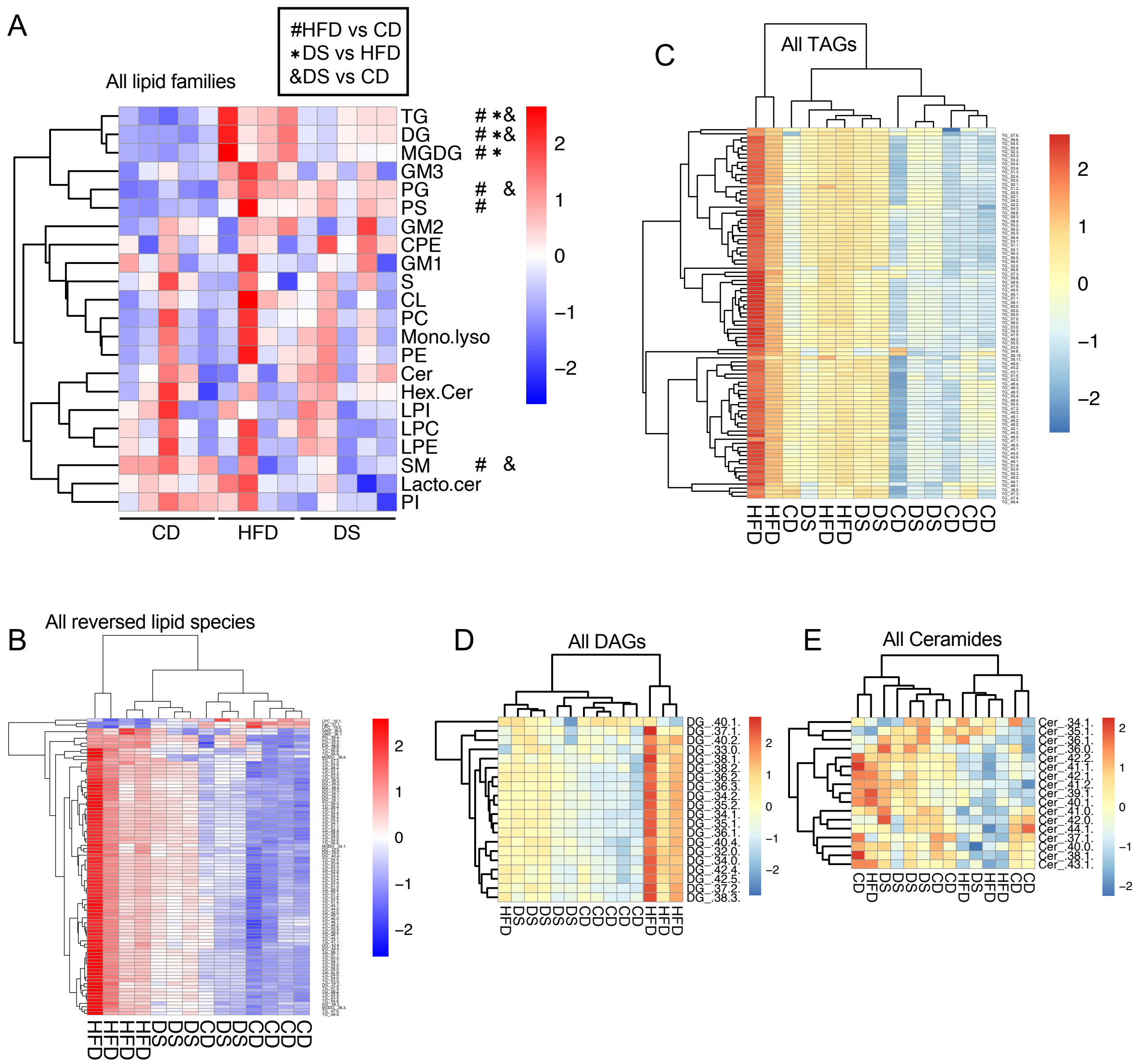

Figure S3. Heatmaps based on lipidomics data from the quadriceps muscle of male mice, related to Figure 3. (**A**) Heatmap showing the total concentrations of each lipid family in CD, HFD fed male mice or those subject to three days DS from HFD to CD. (**B**) Heatmap showing all lipid species that are significantly regulated by HFD feeding compared to CD group and reversed by DS. (**C-E**) Heatmaps showing the concentrations of all TAG (**C**), DAG (**D**), and Ceramide (**E**) species with hierarchical cluster analyses across three diet groups.

The colour gradient for heatmaps represents the z-score values, with red colour indicating higher values and blue indicating lower values. Significance was determined utilizing limma package in R. not significant (ns), p > 0.05, * p < 0.05 between DS and HFD groups, # p < 0.05 between HFD and CD groups, & p< 0.05 between DS and CD groups. (Abbreviations: CD, chow diet; HFD, high fat diet; DS, diet switch; TAG, triacylglycerol; DAG, diacylglycerol)

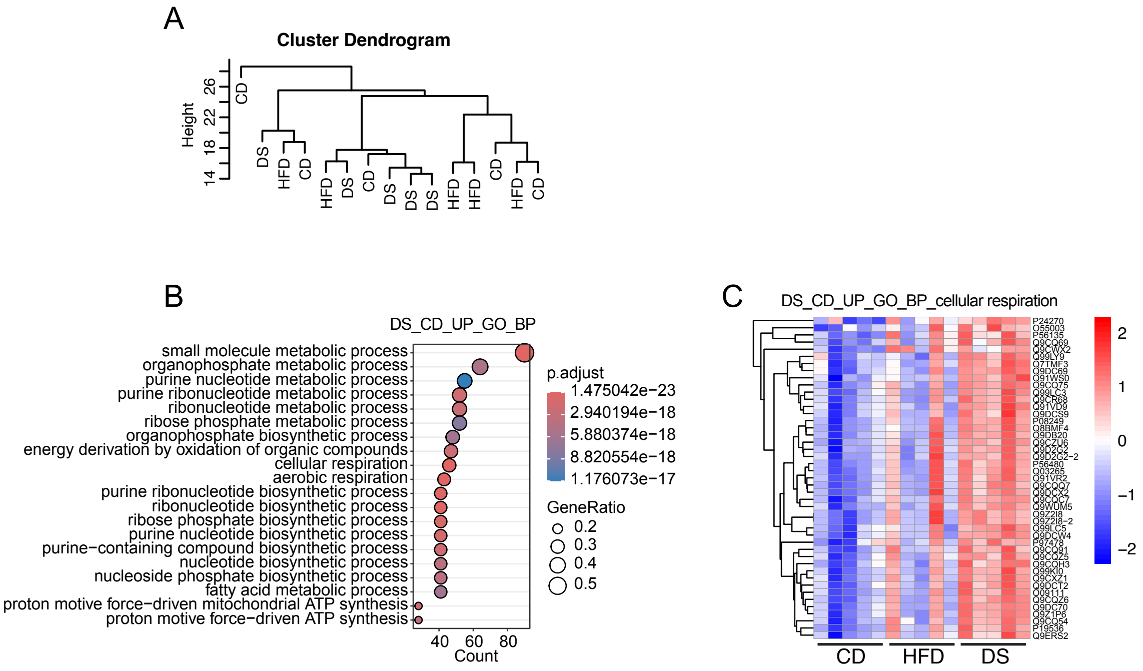

Figure S4. Analyses based on proteomics data from the gastrocnemius muscle of CD, HFD fed male mice or those subject to three days DS from HFD to CD, related to Figure 3. (**A**) Hierarchical cluster analysis. (**B**) Pathway analysis of proteins significantly upregulated in DS group compared to CD group using GO_BP database, top 20 most significantly enriched pathways are listed. (**C**) Heatmap displaying the expression of proteins included in the "cellular respiration" pathway in (**B**).

The colour gradient for heatmaps represents the z-score values, with red colour indicating higher values and blue indicating lower values. (Abbreviations: CD, chow diet; DS, diet switch; GO_BP, gene ontology_biological process)

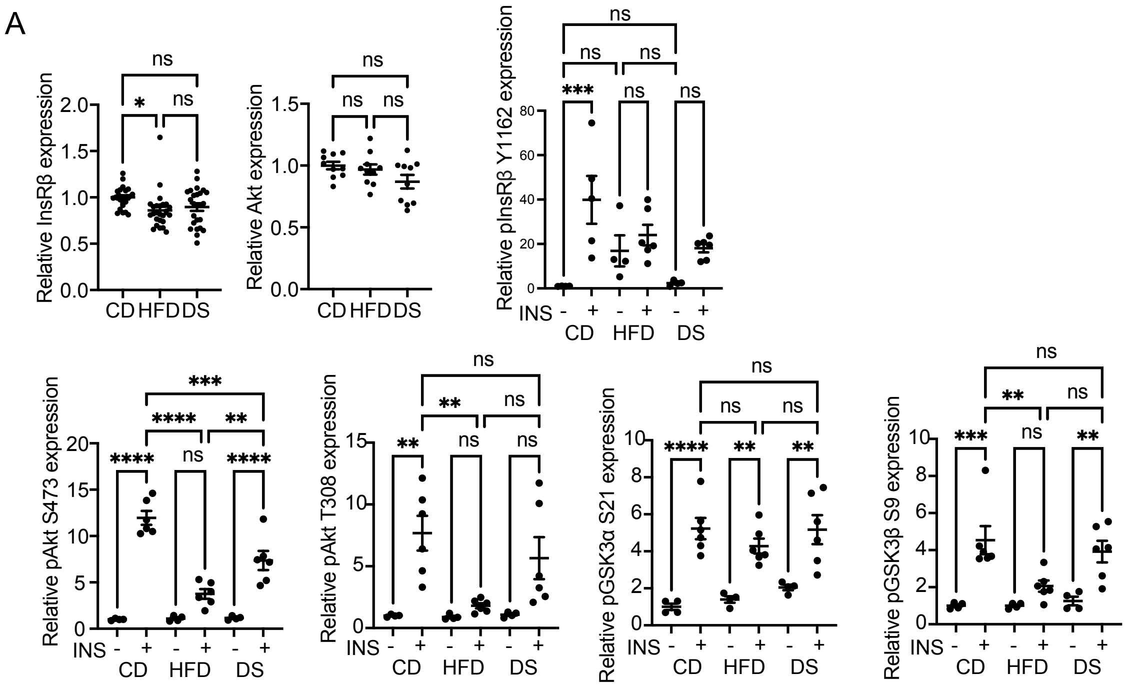

Figure S5. Quantification of western blot data from the liver of male mice, related to Figure 4. (A) InsRβ, Akt, pInsRβ Y1162, pAkt S473, pAkt T308, pGSK3α S21, and pGSK3β S9 expression relative to loading control in the liver from CD, HFD fed mice or those subject to three days DS from HFD to CD (with PBS or insulin injection for protein phosphorylation). Insulin was administered at a dose of 1 U/Kg of the average body weight of CD group mice.

Error bars represent mean ± SEM. Significance was determined using Ordinary one-way ANOVA with Sidak or Tukey test to correct for multiple comparisons. * $p < 0.05$, ** $p < 0.01$, *** $p<0.001$, and **** $p < 0.0001$. (Abbreviations: CD, chow diet; HFD, high fat diet; DS, diet switch)

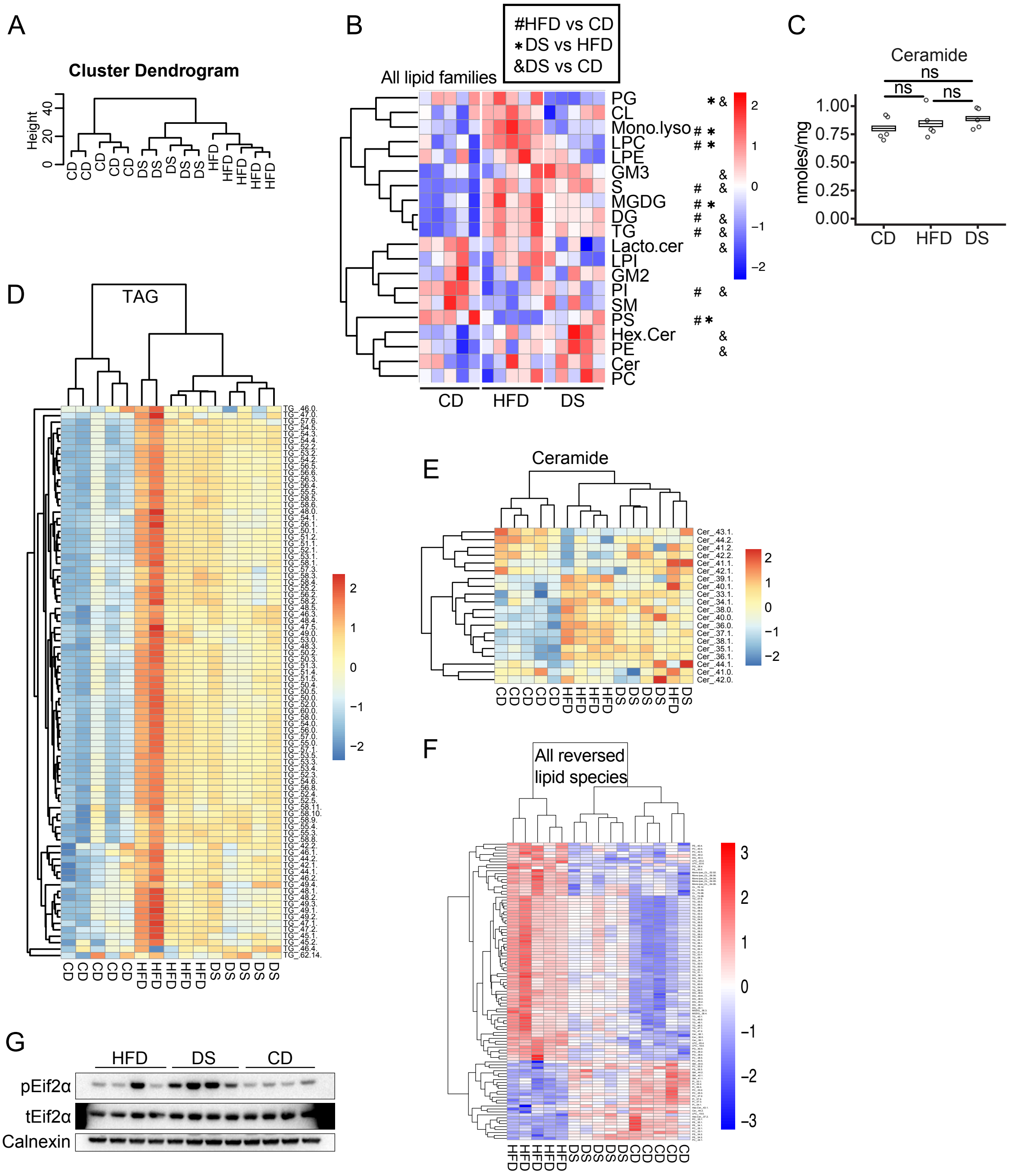

Figure S6. Analyses based on lipidomics data from the liver of CD, HFD fed male mice or those subject to three days DS from HFD to CD, related to Figure 4. (**A**) Hierarchical cluster analysis. (**B**) Heatmap showing the total concentrations of each lipid family in individual mouse from each diet group. (**C**) Concentration of Ceramide in the liver in mice from all diet groups. (**D-E**) Heatmap displaying levels of all TAG (**D**) or Ceramide (**E**) species detected in lipidomics analyses, along with the hierarchical clustering across diet groups. (**F**) Heatmap showing all lipid species that are significantly regulated by HFD feeding compared to CD group and reversed by DS. (**G**) Western blot showing the expression of pEif1α, total Eif2α, and calnexin.

Box plots represent mean ± SEM. The colour gradient for heatmaps represents the z-score values, with red colour indicating higher values and blue indicating lower values. Significance was determined utilizing limma package in R. not significant (ns), $p > 0.05$, * $p < 0.05$ between DS and HFD groups, # $p < 0.05$ between HFD and CD

groups, & p< 0.05 between DS and CD groups. (Abbreviations: TAG, triacylglycerol; CD, chow diet; HFD, high fat diet; DS, diet switch)

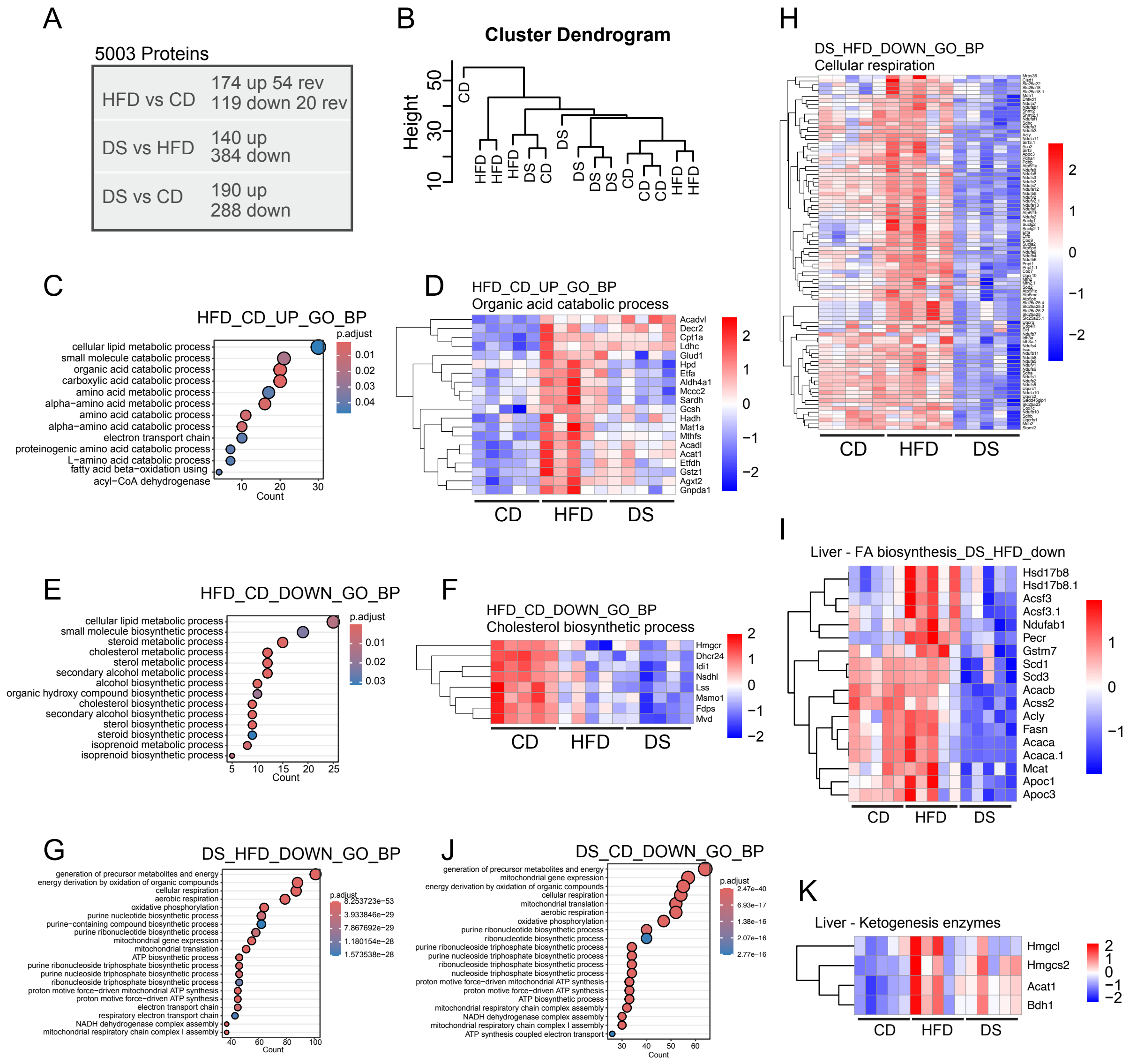

Figure S7. Analyses based on proteomics data from the liver of CD, HFD fed male mice or those subject to three days DS from HFD to CD, related to Figure 4. (**A**) Numbers of significantly regulated proteins (defined as adjusted p-value <= 0.05) in indicated comparisons based on proteomics analyses in the liver. (**B**) Hierarchical cluster analysis. (**C**, **E**, **G**, **J**) Pathway analysis with proteins significantly upregulated by HFD compared to CD group (**C**), proteins significantly downregulated by HFD compared to CD group (**E**), proteins significantly downregulated by DS compared to HFD group (**G**), or proteins significantly downregulated by DS compared to CD group (**J**) using GO_BP database, top 20 most significantly enriched pathways are listed. (**D**, **F**, **H**, **I**, **K**) Heatmaps displaying the expression of proteins included in the "organic acid catabolic process" pathway in (**C**), the "cholesterol biosynthetic process" pathway in (**E**), the "cellular respiration" pathway in (**G**), a FA biosynthesis pathway, or those involved in ketogenesis.

The colour gradient for heatmaps represents the z-score values, with red colour indicating higher values and blue indicating lower values. (Abbreviations: CD, chow diet; HFD, high fat diet; DS, diet switch; FA, fatty acid; GO_BP, gene ontology_biological process)

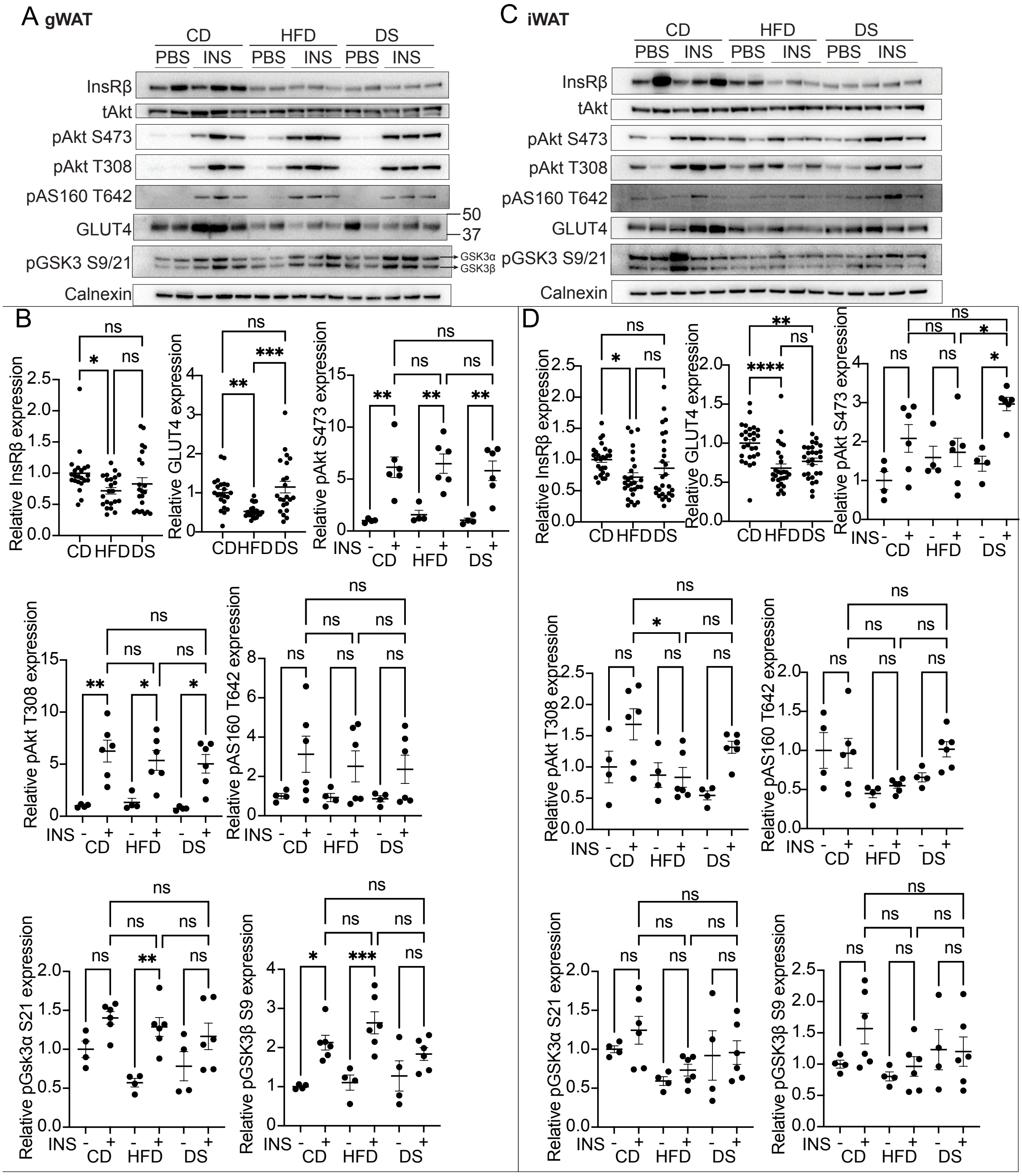

Figure S8. Protein expression of components within insulin signalling pathway in WAT of CD, HFD fed male mice or those subject to three days DS from HFD to CD, related to Figure 5. (**A**, **C**) Western blots of insulin signalling components in gWAT (**A**) or iWAT (**C**) from CD, HFD or DS mice, with or without insulin injection. (**B**) Quantification of (**A**). (**D**) Quantification of (**C**). Insulin was administered at a dose of 1 U/Kg of the average body weight of CD group mice.

Error bars represent mean ± SEM. Significance was determined by Ordinary one-way ANOVA with Tukey (total proteins) or Sidak (phosphorylated proteins) correction for multiple comparisons. not significant (ns), $p > 0.05$, * $p < 0.05$, ** $p < 0.01$, *** $p<0.001$, and **** $p < 0.0001$. (Abbreviations: WAT, white adipose tissue; gWAT, gonadal WAT; iWAT, inguinal WAT; CD, chow diet; HFD, high fat diet; DS, diet switch)

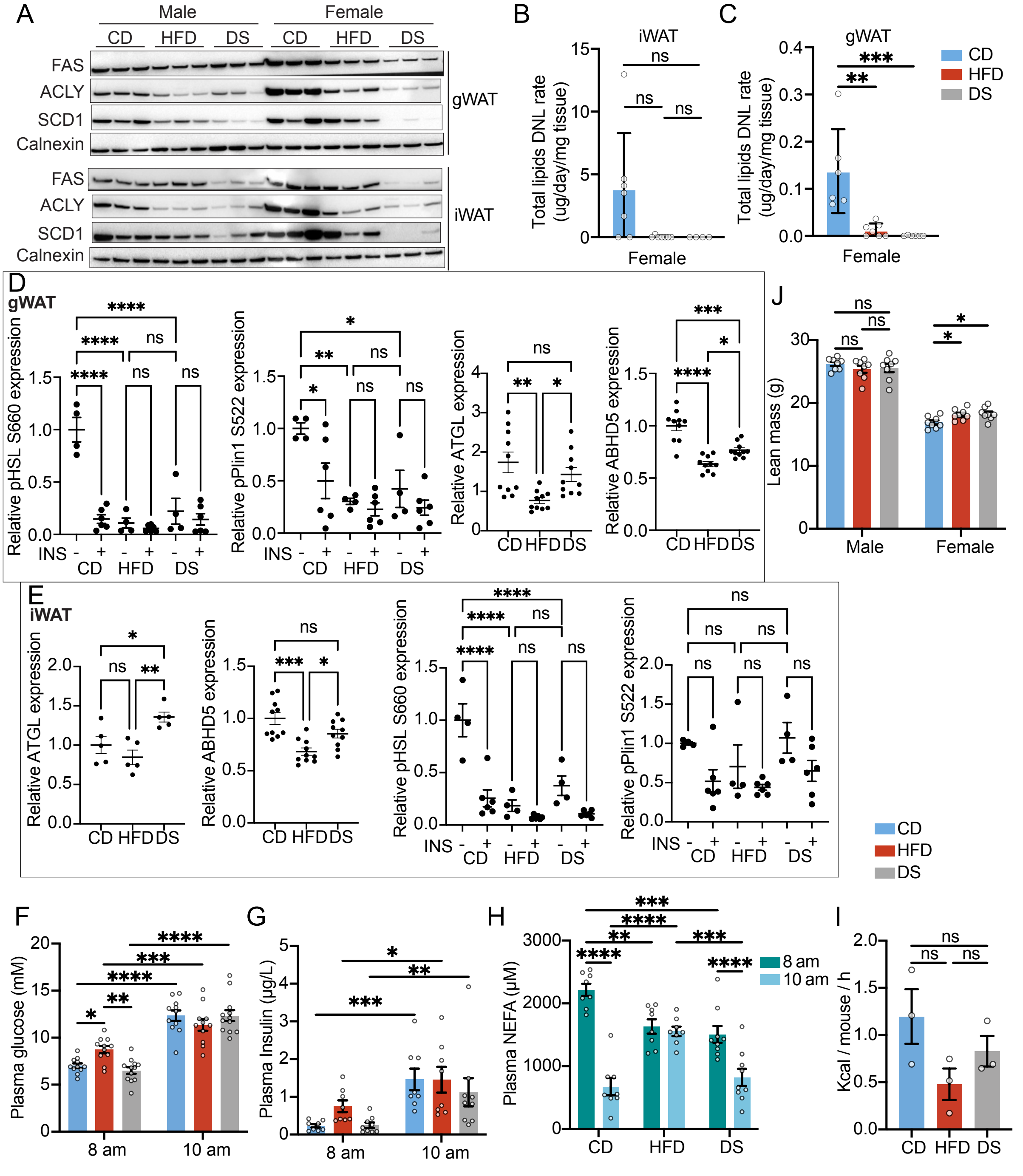

Figure S9. DNL-, and lipolysis-related data in WAT of CD, HFD fed mice or those subject to three days DS from HFD to CD, related to Figure 5. (**A**) Western blots showing the expression of DNL-related proteins, FAS, ACLY, and SCD1 in gWAT and iWAT from male and female mice from CD, HFD and DS groups. (**B-C**) DNL rate in iWAT (**B**) and gWAT (**C**) from female mice. (**D**) Quantification of western blot data from male mice in Figure 5G. (**E**) Quantification of western blot data from male mice in Figure 5H. (**F-H**) Plasma glucose (**F**), plasma insulin (**G**), and plasma NEFA (**H**) from male mice either after overnight fast (8 am time point), or after 2 hours refeeding (10 am time point). (**I**) Food intake of male mice during 2-hour refeeding, the number of dots in the graph represents the number of cages. (**J**) Whole body lean mass of male and female mice fed with CD, HFD, or HFD followed by switching back to CD for three days.

Error bars represent mean ± SEM. Significance for (**B-C**) and (**I-J**) was determined by Ordinary one-way ANOVA with Tukey correction for multiple comparisons. Significance for (**F-H**) was determined by Two-way ANOVA

with Tukey correction for multiple comparisons. not significant (ns), p > 0.05, * p < 0.05, ** p < 0.01, *** p<0.001, and **** p < 0.0001. (Abbreviations: DNL, de novo lipogenesis; WAT, white adipose tissue; gWAT, gonadal WAT; iWAT, inguinal WAT; CD, chow diet; HFD, high fat diet; DS, diet switch; FAS, fatty acid synthase; ACLY, ATP citrate lyase; SCD1, stearoyl-CoA desaturase 1; NEFA, non-esterified fatty acid)

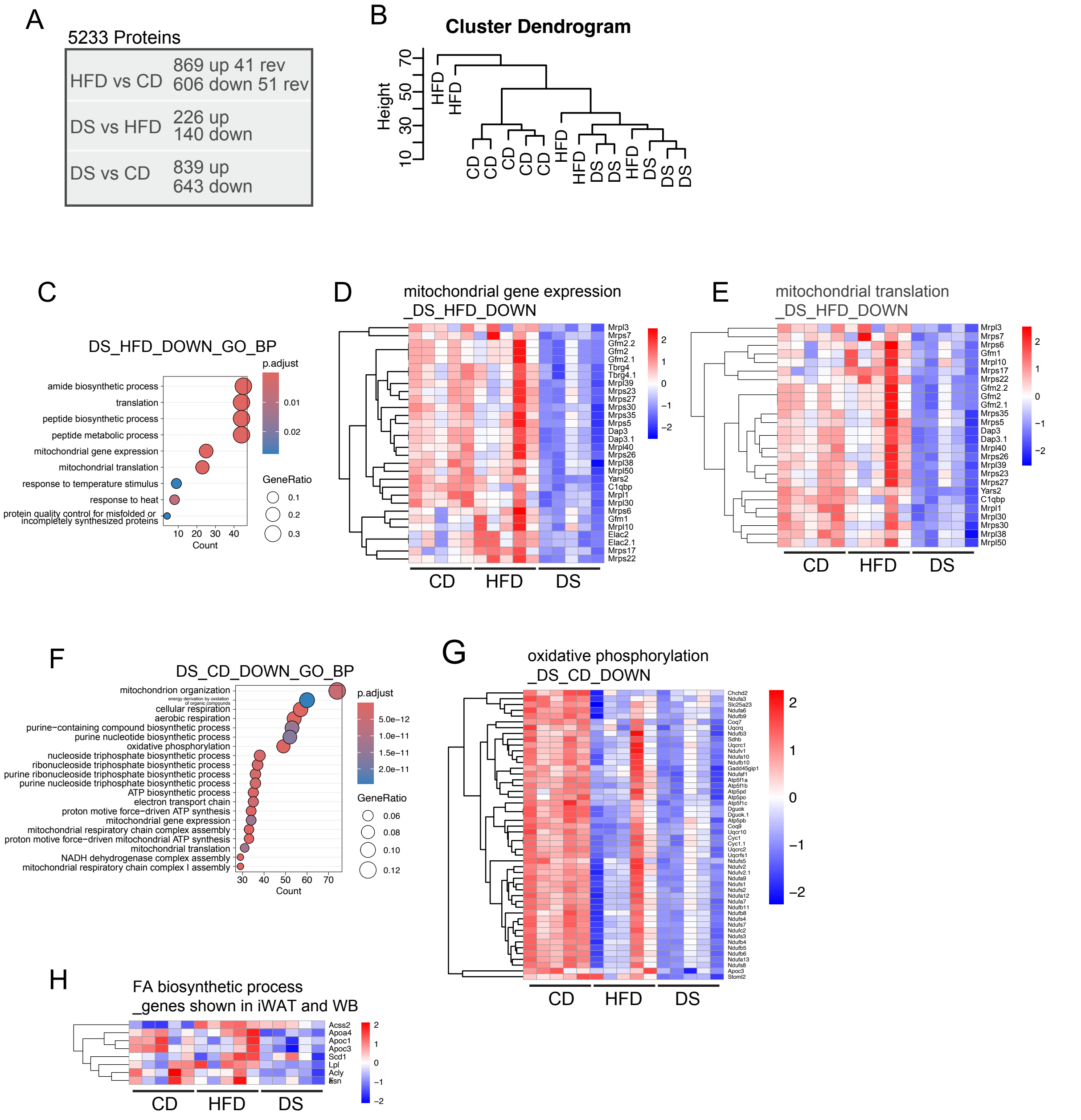

Figure S10. Analyses based on proteomics data from gWAT of CD, HFD fed male mice or those subject to three days DS from HFD to CD, related to Figure 5. (**A**) Numbers of significantly regulated proteins (defined as adjusted p-value <= 0.05) in indicated comparisons based on proteomics analyses in gWAT. (**B**) Hierarchical cluster analysis. (**C**, **F**) Pathway analysis with proteins significantly downregulated in DS compared to HFD group (**C**), or proteins significantly downregulated in DS compared to CD group (**F**) using GO_BP database, top 20 most significantly enriched pathways are listed. (**D-E**) Heatmaps displaying the expression of proteins included in the "mitochondrial gene expression" pathway (**D**) or the "mitochondrial translation" pathway (**E**) in (**C**). (**G**) Heatmap

displaying the expression of proteins included in the “oxidative phosphorylation” pathway in (**F**). (**H**) Heatmap showing the expression of proteins involved in FA biosynthesis as shown in Figure S9A and Figure S11F.

The colour gradient for heatmaps represents the z-score values, with red colour indicating higher values and blue indicating lower values. (Abbreviations: gWAT, gonadal white adipose tissue; CD, chow diet; HFD, high fat diet; DS, diet switch; GO_BP, gene ontology_biological process; FA, fatty acid)

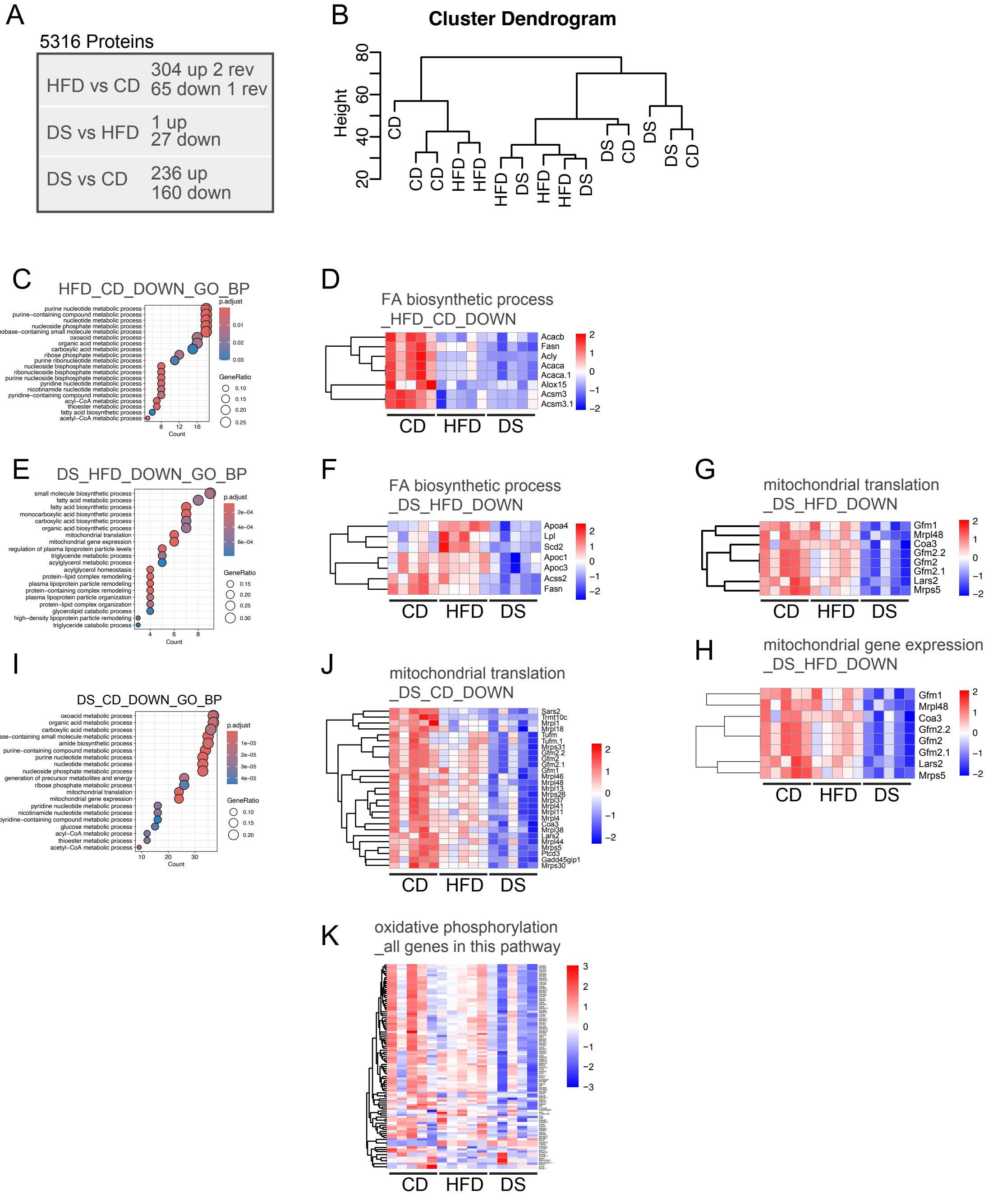

Figure S11. Analyses based on proteomics data from iWAT of CD, HFD fed male mice or those subject to three days DS from HFD to CD, related to Figure 5. (**A**) Numbers of significantly regulated proteins (defined as adjusted p-value <= 0.05) in indicated comparisons based on proteomics analyses in iWAT. (**B**) Hierarchical cluster analysis. (**C,E,I**) Pathway analysis with proteins significantly downregulated in HFD compared to CD group (**C**), proteins significantly downregulated in DS compared to HFD group (**E**), or proteins significantly downregulated in DS compared to CD group (**I**) using GO_BP database, top 20 most significantly enriched

pathways are listed. (**D**) Heatmaps displaying the expression of proteins included in the "fatty acid biosynthetic process" pathway in (**C**). (**F-H**) Heatmaps displaying the expression of proteins included in the "fatty acid biosynthetic process" pathway (**F**), the "mitochondrial translation" pathway (**G**) or the "mitochondrial gene expression" pathway (**H**) in (**E**). (**J**) Heatmap displaying the expression of proteins included in the "mitochondrial translation" pathway in (**I**). (**K**) Heatmap displaying the expression of proteins included in the "oxidative phosphorylation" pathway.

The colour gradient for heatmaps represents the z-score values, with red colour indicating higher values and blue indicating lower values. (Abbreviations: iWAT, inguinal white adipose tissue; CD, chow diet; HFD, high fat diet; DS, diet switch; GO_BP, gene ontology_biological process)

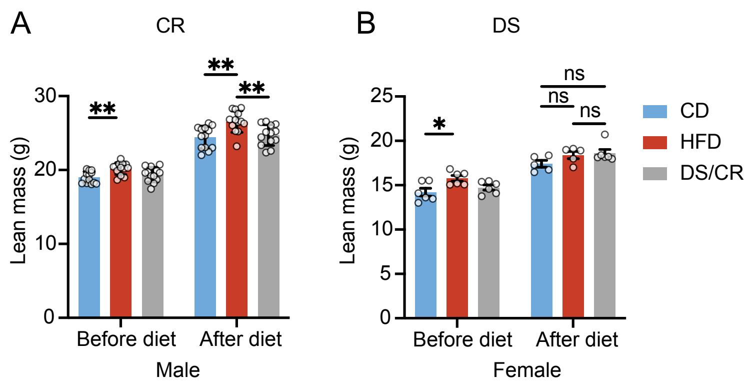

Figure S12. Lean mass of male and female mice, related to Figure 6.

(**A**) Pre- and post-study lean mass of male mice fed with a CD for 18 weeks, or a HFD for 18 weeks, or a HFD for 18 weeks followed by 18 hours of 70% calorie restriction (CR) whilst remaining on a HFD formulation. (**B**) Pre- and post-study lean mass of female mice fed with a CD for 18 weeks, or a HFD for 18 weeks, or a HFD for 18 weeks followed by switching back to CD for three days.

Error bars represent mean ± SEM. n number denotes biological replicates. Significance for (**A**), (**B**) was determined using One-way ANOVA with Tukey test to correct for multiple comparisons. not significant (ns), $p > 0.05$, * $p < 0.05$, ** $p < 0.01$, *** $p<0.001$, and **** $p < 0.0001$. (Abbreviations: HFD, high fat diet; CD, chow diet; DS, diet switch; CR, calorie restriction)
